# Complaints, Complainants, and Rulings Regarding Drug Promotion in the United Kingdom and Sweden 2004–2012: A Quantitative and Qualitative Study of Pharmaceutical Industry Self-Regulation

**DOI:** 10.1371/journal.pmed.1001785

**Published:** 2015-02-17

**Authors:** Anna V. Zetterqvist, Juan Merlo, Shai Mulinari

**Affiliations:** 1 Department of Clinical Sciences, Faculty of Medicine, Lund University, Lund, Sweden; 2 Department of Clinical Sciences, Unit of Social Epidemiology, Faculty of Medicine, Lund University, Lund, Sweden; 3 Department of Sociology, Faculty of Social Sciences, Lund University, Lund, Sweden; Harvard University, Brigham and Women's Hospital, UNITED STATES

## Abstract

**Background:**

In many European countries, medicines promotion is governed by voluntary codes of practice administered by the pharmaceutical industry under its own system of self-regulation. Involvement of industry organizations in policing promotion has been proposed to deter illicit conduct, but few detailed studies on self-regulation have been carried out to date. The objective of this study was to examine the evidence for promotion and self-regulation in the UK and Sweden, two countries frequently cited as examples of effective self-regulation.

**Methods and Findings:**

We performed a qualitative content analysis of documents outlining the constitutions and procedures of these two systems. We also gathered data from self-regulatory bodies on complaints, complainants, and rulings for the period 2004–2012. The qualitative analysis revealed similarities and differences between the countries. For example, self-regulatory bodies in both countries are required to actively monitor promotional items and impose sanctions on violating companies, but the range of sanctions is greater in the UK where companies may, for instance, be audited or publicly reprimanded. In total, Swedish and UK bodies ruled that 536 and 597 cases, respectively, were in breach, equating to an average of more than one case/week for each country. In Sweden, 430 (47%) complaints resulted from active monitoring, compared with only two complaints (0.2%) in the UK. In both countries, a majority of violations concerned misleading promotion. Charges incurred on companies averaged €447,000 and €765,000 per year in Sweden and the UK, respectively, equivalent to about 0.014% and 0.0051% of annual sales revenues, respectively. One hundred cases in the UK (17% of total cases in breach) and 101 (19%) in Sweden were highlighted as particularly serious. A total of 46 companies were ruled in breach of code for a serious offence at least once in the two countries combined (*n* = 36 in the UK; *n* = 27 in Sweden); seven companies were in serious violation more than ten times each. A qualitative content analysis of serious violations pertaining to diabetes drugs (UK, *n* = 15; Sweden, *n* = 6; 10% of serious violations) and urologics (UK, *n* = 6; Sweden, *n* = 13; 9%) revealed various types of violations: misleading claims (*n* = 23; 58%); failure to comply with undertakings (*n* = 9; 23%); pre-licensing (*n* = 7; 18%) or off-label promotion (*n* = 2; 5%); and promotion of prescription drugs to the public (*n* = 6; 15%). Violations that go undetected or unpunished by self-regulatory bodies are the main limitation of this study, since they are likely to lead to an underestimate of industry misconduct.

**Conclusions:**

The prevalence and severity of breaches testifies to a discrepancy between the ethical standard codified in industry Codes of Conduct and the actual conduct of the industry. We discuss regulatory reforms that may improve the quality of medicines information, such as pre-vetting and intensified active monitoring of promotion, along with larger fines, and giving greater publicity to rulings. But despite the importance of improving regulatory arrangements in an attempt to ensure unbiased medicines information, such efforts alone are insufficient because simply improving oversight and increasing penalties fail to address additional layers of industry bias.

## Abstract 2

In a document analysis, Shai Mulinari and colleagues examine the evidence for promotion and self-regulation by the pharmaceutical industry in the UK and Sweden.

## Editors' Summary

**Background:**

Making and selling medicines is big business. In 2013, the global revenue of pharmaceutical companies was nearly US$1 trillion. And every year, a large proportion of this revenue—maybe as much as one-third—is spent on drug promotion. The pharmaceutical companies claim that drug promotion (for example, advertisements in journals and visits from pharmaceutical sales representatives) helps to inform and educate health care professionals about the risks and benefits of medicines. However, drug promotion also has the potential to encourage health care professionals to prescribe inappropriate or needlessly expensive drugs and to encourage the public to buy unnecessary over-the-counter (OTC) drugs (medicines that, unlike prescription drugs, can be bought without a written instruction from a physician or other licensed health care professional). In many countries, including the US, government bodies regulate the promotion of medicines but in other countries, including many in Europe, the pharmaceutical industry self-regulates medicines promotion through voluntary codes of practice.

**Why Was This Study Done?:**

Over the past decade, several whistleblower cases have spotlighted the illicit marketing practices of pharmaceutical companies in the US but relatively few similar cases have been brought in Europe. The reason for this discrepancy is unclear but one possibility is that the wider use of self-regulation in Europe encourages the industry to comply with drug promotion rules and deters illicit conduct. To date, however, self-regulation of medicines promotion has been poorly studied. Here, the researchers undertake a quantitative (numerical) and qualitative (descriptive) study of pharmaceutical self-regulation in the UK and Sweden, two countries often cited as places where self-regulation is effective. In both countries, the rules on medicines promotion are codified in the Code of Practice of the national industry trade group and are overseen by self-regulatory bodies that operate independently of the trade groups; the Swedish code applies to the promotion of both prescription and OTC drugs whereas the UK code applies only to the promotion of prescription drugs.

**What Did the Researchers Do and Find?:**

The researchers first undertook a qualitative content analysis of the documents outlining the rules and procedures governing the self-regulatory bodies overseeing medicines promotion in the two countries. Both bodies, they report, are required to actively monitor promotional items and to impose sanctions on companies that violate the rules. However, a wider range of sanctions, which includes the audit and public reprimand of offending companies in addition to economic sanctions, can be imposed in the UK than in Sweden. Analysis of numerical data collected by the self-regulatory bodies on complaints, complainants, and rulings revealed that between 2004 and 2012 the Swedish and UK bodies ruled that 536 and 597 cases, respectively, were in breach of the country’s rules on medicines promotion; many of the violations in both countries concerned misleading claims about a drug’s effects. In Sweden, nearly half the complaints resulted from active monitoring of promotional items compared to only 0.2% in the UK. Charges incurred by companies because of violations of the medicines promotion code were equivalent to about 0.014% and 0.0051% of annual sales revenue in Sweden and the UK, respectively. Notably, nearly 20% of the cases in breach of the code of practice in both countries were serious breaches, and seven companies were in serious violation more than ten times each in the two countries combined.

**What Do These Findings Mean?:**

These findings indicate that, between 2004 and 2012, there were numerous violations of the pharmaceutical industry codes regulating medicines promotion in both the UK and Sweden. That is, there was a clear discrepancy between the ethical standard codified in the pharmaceutical industry Codes of Practice in these two countries and the actual conduct of the industry. Importantly, the discrepancy may be larger than reported here because the researchers only considered violations that were detected and punished by the self-regulatory bodies in their analysis; some violations that occurred during the study period probably went undetected or unpunished. Given their findings, the researchers suggest that regulatory reforms, including pre-vetting of promotional materials, intensified active monitoring of promotion, larger fines, and the introduction of other sanctions such as greater publicity following rulings, may help to improve the quality of medicines information for health care professionals and the public in Sweden, the UK, and other countries where the pharmaceutical industry self-regulates drug promotion.

**Additional Information:**

Please access these websites via the online version of this summary at http://dx.doi.org/10.1371/journal.pmed.1001785.

- Wikipedia provides information on pharmaceutical marketing (mainly in the US) (note that Wikipedia is a free online encyclopedia that anyone can edit; available in several languages)
- The US Food and Drug Administration Office of Prescription Drug Promotion aims to protect the public health by assuring prescription drug information is truthful, balanced, and accurately communicated; the FDA’s Bad Ad Program aims to educate health care professionals about the role they can play in ensuring that drug advertising and promotion is truthful and not misleading
- Information on the UK regulatory framework is available from the UK Medicines and Healthcare Products Regulatory Agency (MHRA); the MHRA website also includes information on MHRA vetting of advertising material.
- Information on the UK self-regulatory body, the Prescription Medicines Code of Practice Authority (PMCPA), is available; the PMCPA website includes information about the UK Code of Practice for medicines promotion
- Details of the Swedish Code are also available; codes for other European countries are provided by the European Federation of Pharmaceutical Industries and Associations
- Healthy Skepticism is an international non-profit membership association that aims to improve health by reducing harm from misleading health information
- The World Health Organization provides information about drug promotion and attitudes to it in “Drug Promotion—What We Know, What We Have Yet to Learn—Reviews of Materials in the WHO/HAI Database on Drug Promotion”

## Introduction

A string of whistleblower cases in the United States over the past decade has spotlighted the illicit marketing practices of pharmaceutical companies [1,2]. However, in stark contrast to the US, Europe has had few high-profile cases [3]. This invites the question whether illicit marketing is uncommon in Europe, or whether other factors may account for this disparity, such as lack of monetary incentives for whistleblowers, or other aspects of the European legal and regulatory framework. Indeed, it has been suggested that industry may refrain from illicit marketing in Europe because in many countries industry is highly involved in policing marketing claims, which is purported to provide the industry with incentive to comply with rules [4,5]. Thus, in many European countries promotion is governed by a voluntary code of practice administered by the industry’s own system of self-regulation [5–8]. Such an arrangement differs from the US—and some European countries, e.g., France [5]—where regulatory agencies such as the Food and Drug Administration (FDA) directly regulate promotion [9].

Against this background, the present paper analyzes industry self-regulation and medicines promotion in two European countries, the United Kingdom and Sweden, both of which have been lauded as good examples of self-regulation by industry [5,10] and regulators [11,12]. The regulatory arrangements in these countries can best be described as “delegated” self-regulation as an integral part of a co-regulatory scheme involving industry and national medicines regulatory authorities [13]. That is, the medicines regulatory authorities have delegated a significant part of their defined statutory responsibility to the industry trade groups in order to ensure that promotion complies with European Union (EU) and national medicines law.

In the UK and Sweden the rules on medicines promotion are codified in the national industry trade groups’ Code of Practice [14,15]. The Association of the British Pharmaceutical Industry (ABPI) first established the UK Code in 1958. Läkemedelsindustriföreningen (LIF), Sweden’s counterpart of the ABPI, established the Swedish Code in 1969. Since then, both codes have been repeatedly revised and have evolved over time to incorporate principles set out in recommendations by the European and international industry trade associations [16,17], as well as in national and EU law [8]. In addition to setting standards for advertising, the codes have rules governing, among other things, relationships with health professionals and patient organizations. An important distinction is that the UK Code applies solely to promotion of prescription drugs, with over-the-counter (OTC) drug promotion covered by a separate code administered by the Proprietary Association of Great Britain (PAGB, the trade association for manufacturers of OTC medicines and food supplements). By contrast, the Swedish Code lays down standards for promotion of both prescription and OTC drugs, along with veterinary drugs.

**Fig 1:**
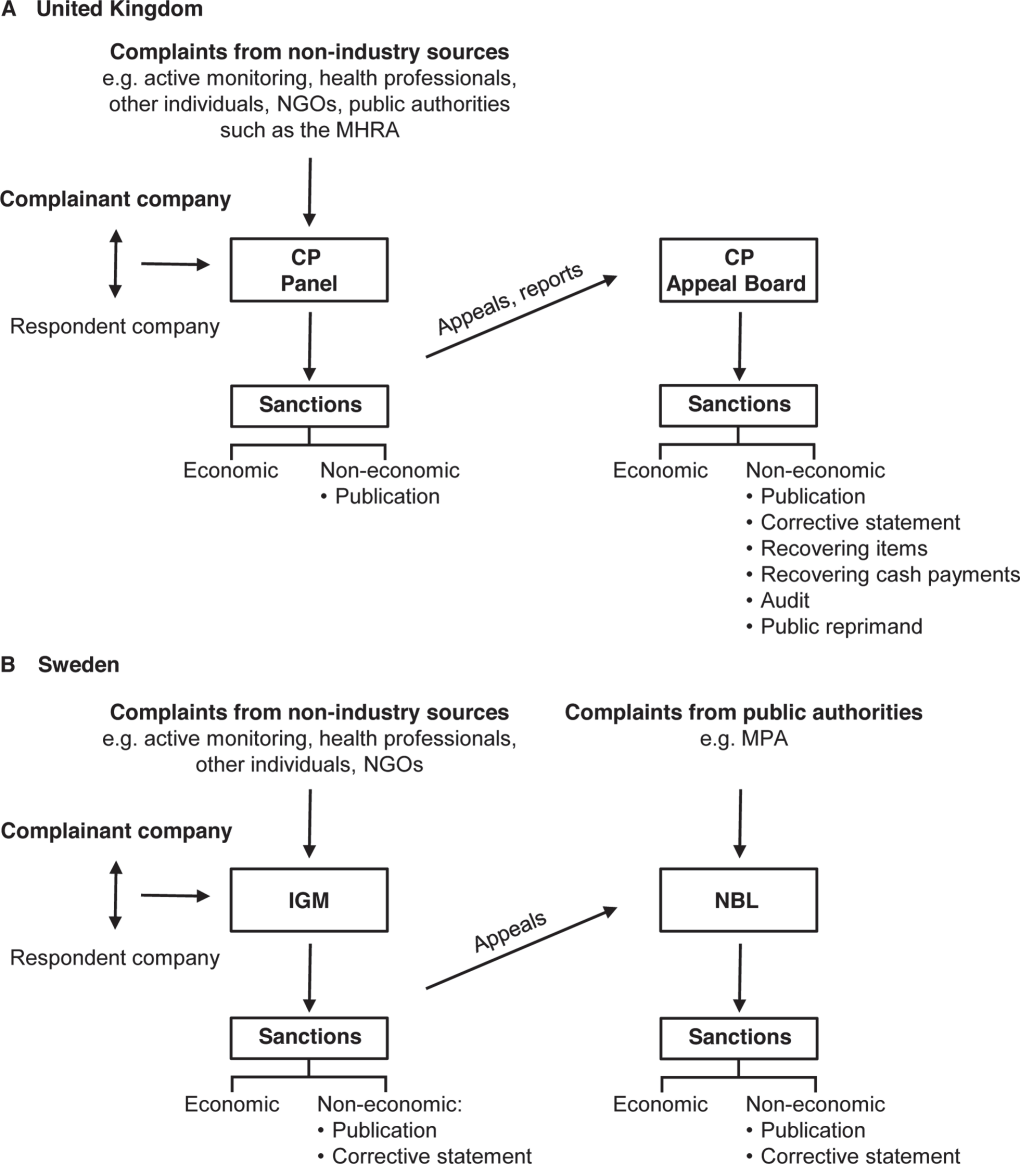
Self-regulatory schemes in the United Kingdom and Sweden. Both countries have a dual-structure self-regulatory system. Complaints emerge from either industry or non-industry sources. In the former case, companies must first attempt to settle disagreements through inter-company dialogue. The Code of Practice (CP) Panel in the UK (A) and the IGM in Sweden (B) impose economic sanctions on violating companies and also publicize rulings. In addition, the IGM may require a company to issue a corrective statement. The CP Appeal Board and the NBL deal with appeals. The CP Panel can also report any company to the CP Appeal Board whose conduct under the Code is of special concern. The CP Appeal Board has a greater range of non-economic disciplinary measures at its disposal than does the NBL. The respective industry trade group may discipline companies in extreme cases (not shown). NGOs, non-governmental organizations.

To enforce the codes, the ABPI and LIF have installed self-regulatory bodies that on a day-to-day basis operate independently of the leadership of the respective associations (Fig. 1). In 1993 the ABPI established the Prescription Medicines Code of Practice Authority (PMCPA) as the quasi-autonomous body responsible for administering the UK Code. The PMCPA consists of the Code of Practice Panel that deals with complaints under the code from whatever source, and the Code of Practice Appeal Board that deals with appeals to the panel’s decisions. The panel can also report any company to the Appeal Board whose conduct under the code is of special concern.

In Sweden, since 1974 two self-regulatory bodies supervise company adherence to the code: the Pharmaceutical Industry’s Information Examiner (IGM) and the Information Practices Committee (NBL). The IGM is appointed to deal with complaints, a role analogous to the UK Code of Practice Panel. However, unlike the Code of Practice Panel, the IGM does not address complaints from public authorities such as the Swedish Medical Products Agency (MPA, the national medicines regulatory authority). Instead, such complaints are sent directly to the NBL committee, which, except in this respect, is functionally analogous to the UK Code of Practice Appeal Board; its main role is to address appeals of IGM decisions.

Despite the significance of pharmaceutical industry self-regulation, this process has received limited scrutiny, perhaps reflecting a US bias in the literature on drug promotion and its regulation. The few existing empirical studies have nevertheless suggested that ethical standards are usually weak, enforcement is ineffective, and sanctions are mild [18–21]. Admittedly, however, the limited number of empirical studies in conjunction with the evolving nature of regulatory systems militates against definitive conclusions. Moreover, differences among self-regulatory schemes could make it problematic to extrapolate findings from one country to another [19]. The latter point underscores the need for international comparisons, which have been successfully carried out in other areas of pharmaceutical regulation [22]. Such comparisons could serve as the basis for suggestions to reform regulatory arrangements in an effort to improve the quality of medicines information in the interest of public health.

With that in mind, our purpose was 2-fold. First, we sought to compare self-regulation in the two European countries in order to identify regulatory strengths and weaknesses. Second, we sought to investigate discrepancies between the industry codes of conduct and the actual practices of companies. For both purposes we investigated documents and case reports issued by self-regulatory bodies in the UK and Sweden.

## Methods

### Study Overview

This study investigates industry self-regulation and promotion of pharmaceuticals for human use in the UK and Sweden. In a first step, we used a qualitative approach to outline similarities and differences between systems regarding constitution and procedures. In a second step, we collected data from both systems regarding various parameters, including registered complaints, rulings, complainants, and specific clauses in the code cited in rulings of breach. These data offer insights into the workings of self-regulatory systems, but also into the violative promotional activities of companies. Much of the UK data are published in annual PMCPA reports. One caveat is that data are currently only available for 2004–2012. We therefore restricted our study to this interval. In a third step, we examined the sanctions imposed by self-regulatory bodies on companies and used these figures to estimate the overall financial cost to manufacturers associated with sanctions. In a fourth and final step, we used violations highlighted as particularly serious by self-regulatory bodies to investigate the prevalence and nature of violation rulings considered especially grave in each country. All quantitative data (e.g., the number of complaints and rulings of breach, and clauses cited in rulings of breach) were retrieved and coded independently by two authors (SM and AVZ). In cases of discrepancy between authors’ assessments, a consensus decision was reached following a joint retrieval and coding of the data.

### Qualitative Analysis of Self-Regulatory Systems

We conducted a systematic analysis of documents detailing the constitution and procedures of self-regulation in the UK and Sweden. This includes the respective codes and associated documents outlining the workings of the systems, but also reports produced by self-regulatory bodies on an annual, biannual, or quarterly basis, as well as individual case reports issued by self-regulatory bodies (Table 1). This material is publicly available on the PMCPA and LIF web sites. Relevant analytic categories were generated by one of the authors (SM) using qualitative content analysis whereby a body of text under scrutiny is condensed into coding units representing various categories and sub-categories (S1 Text) [23].

**Table 1:** Information from self-regulatory bodies in the UK and Sweden.

Reports	United Kingdom	Sweden
Case reports	Individual reports of all cases subject to complaint are publicly available on the PMCPA website. Cases searchable based on words or phrases.	Individual reports of all cases subject to complaint are publicly available on the LIF website. Cases searchable based on words, phrases, company, ATC Code (at any level), brand name, and clause.
Information in case reports	Besides detailing the case, reports include a case number ID and information on the complainant, respondent and the medicine(s) (typically brand names) and the clauses and sub-clauses breached or allegedly breached.	Besides detailing the case, reports include a case number ID and information on the complainant, respondent and the medicine(s) (typically brand and generic names and ATC codes) and the clauses breached or allegedly breached
	Case reports contain no explicit information on administrative charges.	Case reports contain explicit information on administrative charges
	Case reports do not typically contain violating material.	Case reports typically provide the violating material as PDF file
	Case reports specify all claim(s) in breach of the code, but do not indicate all publications or activities where these claims were made prior to the ruling.	Case reports specify all claim(s) in breach of the code, but do not indicate all publications or activities where these claims were made prior to the ruling
Information in summary reports	Quarterly reports with detailed reports of cases and a summary of rulings	Biannual IGM reports with statistics on complaints and rulings
	Annual reports with statistics on complaints, rulings, and accounts	Annual NBL reports with limited statistics. No information on accounts

### Quantitative Analysis of Industry Self-Regulation

**Complaints and rulings.** We compiled longitudinal data on registered complaints and cases ruled in breach. For the UK, we also gathered data on the individual matters found to be in breach, since in the UK (though not in Sweden) each case is subdivided into multiple matters for independent rulings. UK data were obtained directly from the PMCPA annual reports. For Sweden, data pertaining to the IGM are available in a supplement to the biannual IGM reports, which are available upon request from the LIF. Data pertaining to the NBL since 1991 are available on a yearly basis in annual reports. Unlike the UK Code, the Swedish Code also applies to promotion of veterinary products. The IGM/NBL database is searchable using Anatomic Therapeutic Chemical (ATC) classification system codes, and cases pertaining to veterinary products were identified through the ATC code (begins with Q) and excluded from the analysis. Similarly, breaches subject to appeal were subtracted from the total if the appeal was successful.

**Complainants.** The PMCPA annual reports, the IGM supplement to the biannual reports, and the NBL annual reports also contain information on the number of complaints by type of complainant. In each case, data are presented numerically for specific complainant categories. We employed the following categories that are used in both countries: industry, health professionals, Swedish MPA, or UK Medicines and Healthcare Products Regulatory Agency (MHRA, the national medicines regulatory authority), other organizations or bodies (e.g., UK National Institute of Clinical Excellence, Sweden’s Dental and Pharmaceutical Benefits Agency), active monitoring by self-regulatory bodies of promotional material after dissemination (so-called *ex-post* scrutiny), and others (e.g., anonymous, member of public, industry employee or ex-employee). For the UK, we also included the category “other PMCPA Director.” These are complaints nominally attributed to the PMCPA Director but not initiated following active monitoring. Instead, such complaints have usually emerged either in response to media criticism, from voluntary admissions by companies, or when there is an alleged breach of undertaking.

**Active monitoring by self-regulatory bodies.** The number of cases taken up following active monitoring in the UK is reported under a separate heading in the PMCPA annual reports. In Sweden, the IGM is responsible for active monitoring. This work is detailed in a supplement to the biannual IGM reports, which contains data on the number of scrutinized objects by type (i.e., journal ads to health professionals, internet ads, ads to the public, mailings to health professionals).

**Pattern of code breaches.** For each case in Sweden and each matter in the UK, self-regulatory bodies can issue rulings of breach of one or more clauses in the code. Because each case in the UK may involve numerous matters (see above), the number of rulings of breach of specific clauses is far higher in the UK. This difference is augmented by the fact that UK self-regulatory body rulings specify sub-clauses (e.g., two breaches to §7.2; two breaches to §7.3), whereas Swedish self-regulatory bodies typically specify only the breached clause (e.g., breach to §7).

UK information on breached clauses was collected on a case-by-case basis from quarterly reports that include a summary of the outcomes for each case addressed by the PMCPA. For Sweden, data pertaining to breaches of specific clauses identified by the IGM since 1998 are available as supplements to the IGM biannual reports. Although information on breaches is not summarized in the same way for NBL rulings, we compiled this information by reviewing all such rulings in the database for 2004–2012 and recorded the specific clauses breached on a case-by-case basis. We subtracted breaches subject to successful appeal or pertaining to veterinary products (identified as above). Notably, both the UK and Swedish Code has undergone some changes since 2004. Our coding follows the 2012 version of the Codes.

**Economic sanctions and cost calculations.** Self-regulatory bodies may impose economic sanctions on violating companies. Arguably, for economic sanctions to act as a deterrent they cannot be negligible in proportion to industry revenues. We therefore analyzed the charges paid by the industry both in absolute amounts (in EUR) and in relation to total revenues from drug sales. Economic sanctions were defined as the charges imposed on companies by self-regulatory bodies and also, in the UK where this is applicable, as costs for audits and corrective ads or public reprimands paid by violating companies. We estimated industry revenues using annual sales of drugs at manufacturers’ prices, i.e., excluding pharmacy margins. For the UK, we considered sales of prescription drugs, while for Sweden we considered sales of both OTC and prescription drugs since the Swedish Code applies to both. UK sales data at manufacturers’ prices through 2011 were obtained from [24] and are based on a standard manufacturers’ discount rate to wholesalers of 12.5%. We estimated the 2012 value by adding +1.5% to the 2011 value in accordance with available estimates of the increase in cost of medicines to the UK National Health Service, the publically funded health care system, in 2012 [25]. Swedish sales data on manufacturers’ prices are not available. However, we estimated this figure by applying a correction factor of −17.4% to total sales [26] in order to arrive at an estimate of pharmacy margins, as was suggested by personnel at the Swedish Dental and Pharmaceutical Benefits Agency (personal communication, E. Ahlo to SM), which determines what therapeutic products and devices will be state subsidized.

The PMCPA annual reports contain data on yearly economic sanctions imposed on violating companies. Swedish data are not similarly publicized. However, upon request, LIF personnel provided us with data from 2009 to 2012, but informed us that data prior to 2009 were unavailable. For Sweden, we arrived at the totals used to calculate economic sanctions by subtracting any charges for veterinary drug promotion for each year, as specified in case reports identified according to the procedure described above. To convert SEK and GBP into EUR, respectively, on an annual basis we used the average annual exchange rates available from the Swedish Riksbank [27] and the Bank of England [28].

**Non-economic sanctions.** In addition to economic sanctions, self-regulatory bodies may impose non-economic sanctions other than the standard publication of case reports, such as requiring companies to issue corrective statements. UK data on non-economic sanctions were collected on a case-by-case basis from quarterly reports. For Sweden, we did not encounter any references to non-economic sanctions in the IGM biannual or NBL annual reports (other than the publication of case reports). We therefore contacted the LIF, which provided us with the relevant information.

### Serious Violations of the Industry Codes

Both self-regulatory systems have ways to highlight what they consider to be particularly serious violations. In the UK, this is done by ruling of breach of §2 (promotion that “brings discredit to, and reduction of confidence in, the industry”) and/or by imposing non-economic sanctions other than the publication of a case report. The Swedish Code does not contain an equivalent of the UK §2. However, because a differentiated rate has been applied in Sweden since 2004 for simple, common and serious offences, with the highest rate category (SEK 100,000, approximately €11,200, or more) reserved for serious offences, it is nonetheless possible to identify rulings involving serious violations. The charge levied in each ruling is highlighted in the IGM/NBL database and by chronologically browsing through the database we were able to identify rulings of serious violations for each year. Again, we excluded cases subject to successful appeal or pertaining to veterinary products.

We also investigated which clauses were cited in rulings and what companies, complainants, and drugs were involved. We derived this information from each case report. Again we used the 2012 version of the codes for our analysis. Regarding drugs, we coded data using ATC codes. A few (*n* = 9) UK cases involved more than one §2 ruling and these were coded as multiple violations. When compiling data on sanctioned companies we used the ABPI and LIF lists of existing member and affiliate companies available on the websites of the respective organizations. In cases where a sanctioned company was not listed we checked whether that company had merged with or been acquired by another company (e.g., Wyeth by Pfizer). In such cases we assigned the breach to the existing company (i.e., Pfizer). In cases where the violation was by a subsidiary company (e.g., McNeil) we assigned the breach to the parent company (i.e., Johnson & Johnson).

To further investigate illicit promotion, we focused on the therapeutic class associated with the highest number of rulings of serious breaches in each country. We reasoned that this would provide a reasonable number of cases for in-depth analysis without introducing a country bias. Each case report was read, summarized, and then coded [23] according to type(s) of marketing violation (e.g., misleading claim, off-label promotion) by one of the authors (SM) based on the compiled summary. The summary and coding were checked for accuracy against the case reports by another author (AVZ).

### Data Processing and Analysis

We used descriptive analysis to explore trends in complaints, rulings of breach, and statutory copies of promotional material submitted by companies to the IGM for active monitoring (in the UK companies are not required to submit material, see below). We used the number of statutory copies as an indicator of marketing activity in Sweden. Descriptive analysis was also used to analyze other parameters, e.g., clauses in the code cited in rulings and complainants. Data were analyzed using GraphPad Prism 6.0 (GraphPad software).

## Results

### Constitution and Procedures of Self-regulation in the UK and Sweden

Table 2 compares the UK and Sweden systems across some pertinent constitutional and procedural characteristics. It is the industry trade groups that install the self-regulatory bodies. Thus the ABPI appoints the Code of Practice Panel, which comprises a director, deputy director, secretary, and deputy secretary, as well as the Appeal Board, which comprises a legally qualified chairperson and 15 members. While eight members of the Appeal Board represent industry, the remaining eight represent other interests: three medical practitioners, one pharmacist, and one nurse prescriber—appointed following consultation with the British Medical Association, Royal Pharmaceutical Society, and Royal College of Nursing, respectively—along with one patient advocate, one lay member, and one representative from an independent body providing medicines information—all appointed following consultation with the MHRA.

**Table 2:** Constitution and procedures of self-regulation in the UK and Sweden.

Constitution and Procedures	United Kingdom	Sweden
Scope of code	Promotion of prescription drugs. Applies to members of the ABPI. Non-ABPI members may agree to abide by the Code.	Promotion of prescription, OTC, and veterinary drugs. Applies to members of LIF, and the trade groups for small life science companies (IML) and generic manufacturers (FGL).
Bodies	PMCPA: Panel and Board of Appeal. See text for details.	IGM and NBL. See text for details.
Financing	Administrative charges, levies from ABPI members and seminar fees	Administrative charges and funds from LIF
Pre-vetting	No	IGM pre-vets vaccination programs and drug information on company websites aimed at the public.
Active monitoring by self-regulatory bodies	Yes. See text for details.	Yes. See text for details.
	If a company accepts a ruling of breach, and agrees to stop dissemination of material, no charges will be applied and there will be no case report.	If a case is of minor importance, and the company has terminated the practice, or immediately rectifies the matter, the case may be dismissed and there will be no case report.
Sanctions	See Fig. 1.	See Fig. 1.
Public information release	Announcements in professional literature including details of cases where companies are ruled in breach of §2 (see text), or are required to issue corrective statements or are the subject of public reprimand.	No policy on advertising violations
Rules on complaints procedure	Inter-company dialogue required	Inter-company dialogue required
	Anonymity of complaining individuals as a rule	No anonymity of complainant as a rule
	If a company voluntarily reports a possible breach, and is subsequently ruled in breach, charges will be reduced by half.	No policy on voluntary admission of breach
Rule on cases pending appeal	If the panel considers that the material or activity is likely to prejudice public health and/or patient safety, and/or that it represents a serious breach, it must decide whether use of the material or activity should be suspended pending final outcome of the case.	Irrespective of whether an appeal has been lodged, companies must comply with IGM requests until the NBL decides otherwise.

The Swedish IGM is a scientifically qualified physician. As of 2013, two IGMs may be appointed by the LIF: one responsible for activities and material geared toward health professionals, the other for activities and material geared toward the public. The NBL committee consists of a legally qualified chairperson and 11 members: six represent industry, three the general public, and two are medical experts. The LIF appoints the entire committee; however, the representatives of the general public and medical experts are appointed following consultation with an appropriate body or authority representing consumers and the Swedish Medical Association, respectively.

Notably, the rules of procedures specify that self-regulatory bodies in both countries should not only consider lodged complaints, but also undertake routine active monitoring of promotional items in relation to requirements stipulated by the Code. In Sweden, companies are obliged to submit statutory copies of promotional print material, mailings, Internet ads, and films to the IGM to facilitate monitoring. In the UK, however, companies are only obliged to submit material upon request.

Another important procedural feature in both countries is that companies must first attempt to settle disagreements through inter-company dialogue; the complainant company should only lodge a complaint for arbitration if such efforts should be unsuccessful (Fig. 1). By contrast, non-industry complainants directly lodge complaints with self-regulatory bodies. In Sweden, an exception to the inter-company dialogue requirement may be permitted “if the measure constitutes a serious disregard of good industrial practice or if a prompt intervention is required to prevent further damage caused by the measure” (p. 69) [14]. In the UK, an exception is made “where the allegation is that a company has failed to comply with an undertaking that it has given” in relation to a previous ruling (p. 44) [15].

As outlined in Fig. 1, the non-economic sanctions available to self-regulatory bodies differ somewhat between these two countries. A shared feature, however, is that any economic sanctions imposed on companies are collected as a contribution to the self-regulatory system. In cases of violation, the sanctioned company pays administrative charges; however, if a complaint brought by one company against another company is ruled invalid, the complainant company pays the administrative charge. Non-industry complainants are exempt from this rule. In the UK, companies pay on the basis of the number of matters ruled upon; numerous matters may arise in a particular case and each is considered independently. Currently, the administrative charge per matter for an ABPI member that accepts the Panel’s ruling is about €3,600, and the charge per unsuccessfully appealed matter is about €13,200. This differs from Sweden, where each complaint is considered to be a single entity and where a differentiated rate has been applied since 2004, currently about €4,500, €10,200, and €15,800 for simple, common, and serious offences, respectively. Higher charges may be imposed for breaches of undertaking, with a ceiling of about €52,500. There is also an extra charge of about €4,500 for unsuccessfully appealed cases.

### Complaints and Rulings of Breach of Industry Codes 2004–2012

**Fig 2:**
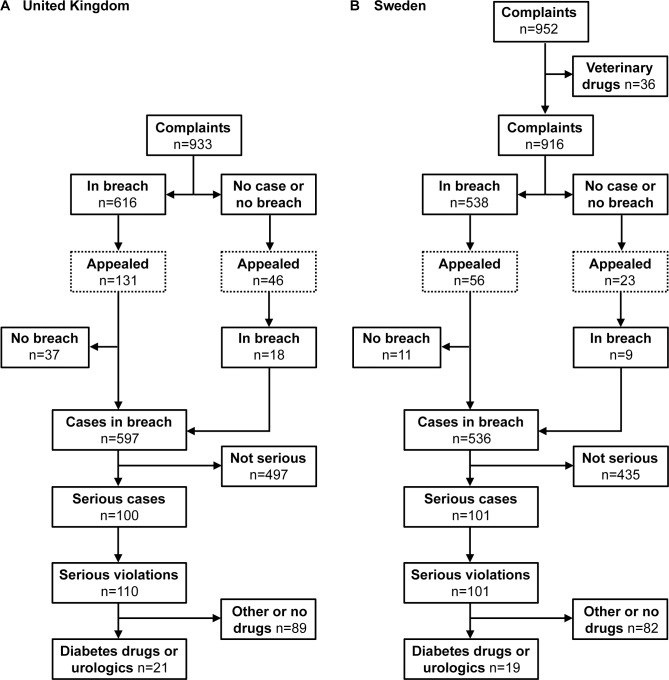
Flow diagram of selected complaints, cases, and violations in the United Kingdom (A) and Sweden (B) 2004–2012.

**Fig 3:**
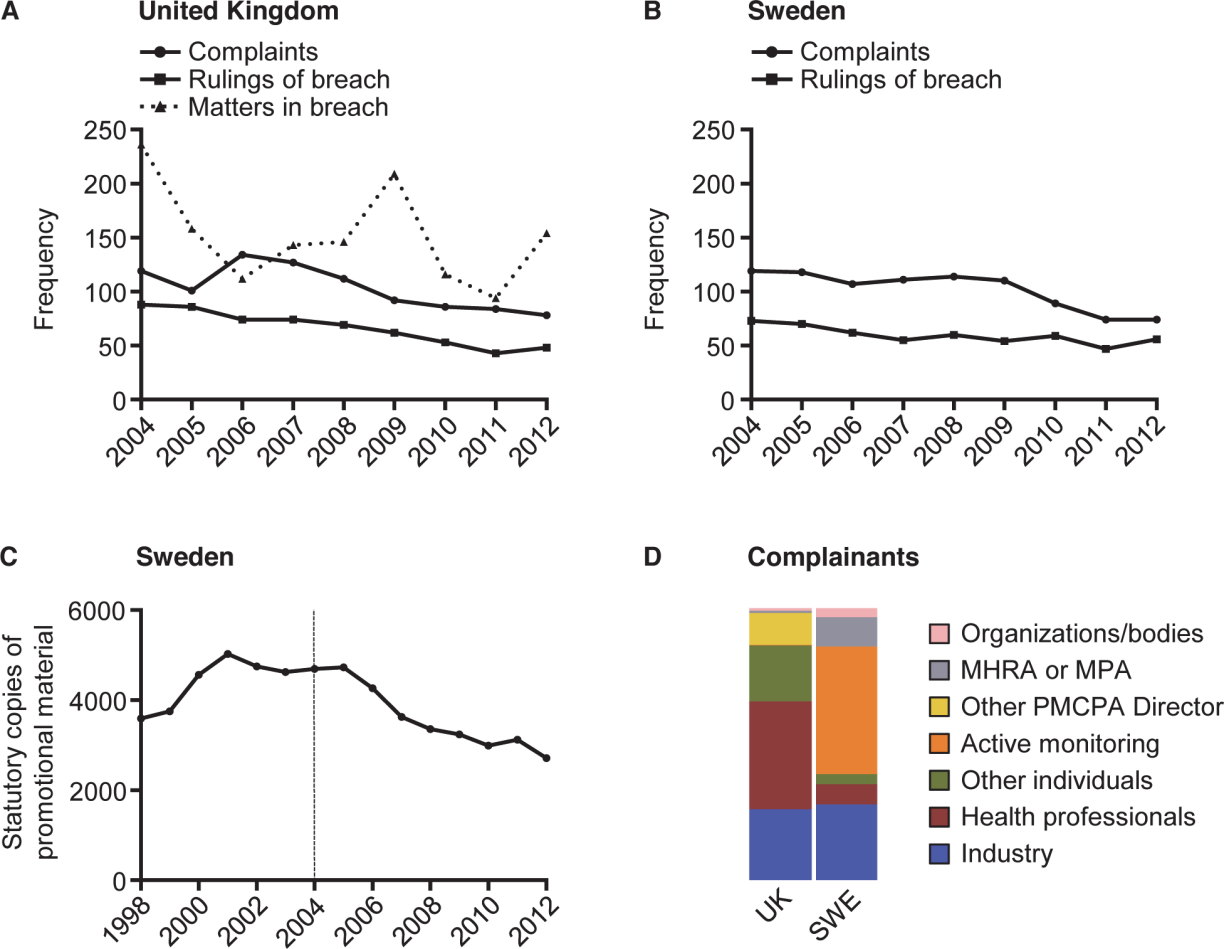
Complaints, rulings of breach, and complainants in the United Kingdom and Sweden 2004–2012. (A) and (B) show the number of registered complaints and cases ruled in breach in the UK and Sweden, respectively (total registered complaints: *n* = 933 in UK, *n* = 916 Sweden; total cases ruled in breach: *n* = 597 in UK, *n* = 536 in Sweden). (A) also shows matters in breach in the UK (*n* = 1368). (C) Statutory copies of drug promotion sent by companies in Sweden to the IGM for scrutiny in 1998–2012 (*n* = 59,158). Dashed line indicates year 2004. (D) shows mosaic plot of registered complaints by type of complainant in each country.

The study selection process is shown in Fig. 2. The numbers of complaints and cases ruled in breach on a yearly basis were similar in both countries: median number of complaints in the UK was 101 (range 78–134) and cases ruled in breach 69 (range 43–88), and in Sweden 110 (range 74–119) and 59 (range 47–73), respectively. The number of complaints and cases ruled in breach appear to have decreased over the studied period in both countries (Fig. 3A and 3B). The average annual decline in complaints in the UK was −5.1 (standard deviation [SD] = 16.7) and in cases ruled in breach −5.0 (SD = 5.7), and in Sweden −5.6 (SD = 9.1) and −2.1 (SD = 7.5), respectively. However, the number of individual matters in breach in the UK (not applicable in Sweden) varied substantially, with no obvious decrease over time. In Sweden, the decrease in complaints and violation rulings coincided with a drop in the number of statutory copies submitted by companies for scrutiny (from 4,693 in 2004 to 2,715 in 2012; average annual decline −247 [SD = 247]) (Fig. 3C). From 2004 to 2012, violation rulings in Sweden decreased with 23% but statutory copies decreased with 41%. The decrease in statutory copies is largely explained by a more than 3-fold decrease in the number of journal advertisements aimed at health professionals (from 1962 in 2004 to 598 in 2012; S1 Fig.).

### Complainants

Fig. 3D shows the source of the complaints. Four findings are readily apparent from this figure. First, the proportion of complaints from industry is similar in both countries: 26% (*n* = 243) in the UK and 28% (*n* = 255) in Sweden. Second, the Swedish MPA regularly lodges complaints, while the UK MHRA does not; indeed, the MHRA only lodged seven complaints (0.8% of total), but there were 99 MPA complaints (11%). Third, the number of cases initiated following complaints from health professionals and other individuals is much larger in the UK (*n* = 370, 40% and *n* = 192, 21%, respectively) than in Sweden (*n* = 68, 7.4% and *n* = 34, 3.7%, respectively). Interestingly, we found a number of anonymous and non-anonymous complaints in the UK from industry employees/ex-employees (*n* = 22, 2.4% and *n* = 23, 2.4%, respectively), but none in Sweden.

Fourth, the IGM, unlike the PMCPA Director, regularly initiates cases following active monitoring. As a result of this monitoring, the IGM initiated 430 cases (47% of total). By comparison, the PMCPA Director initiated two cases (0.2%) following active monitoring, one in 2004 and one in 2006. In addition, from 2006 to 2012 the PMCPA Director reported that 25 advertisements were under scrutiny for potential breach of the Code and that all cases were resolved administratively with the companies involved (no data were available for 2004 and 2005). Only one of these cases was addressed in 2009 and none between 2010 and 2012.

### Pattern of Code Breaches

In the UK, there were 1950 rulings of breach of specific clauses over the study period (Table 3 for selected clauses; S1 Table for complete list). Over 50% (*n* = 1,021) pertained to §7, i.e., “information, claims and comparisons”; of those, over half (*n* = 558) concerned §7.2 mandating that information, claims, and comparisons must be accurate, balanced, fair, objective, and unambiguous, must reflect an up-to-date evaluation of all the evidence and must not mislead. Notably, 5.8% (*n* = 113) of rulings concerned pre-licensing promotion or promotion inconsistent with the terms of the marketing authorization or the summary of product characteristics (SPC) (§3), 4.9% (*n* = 96) concerned conduct of industry representatives (§15), 4.3% (*n* = 84) concerned promotion of prescription drugs to the public (§22), and 3.7% (*n* = 73) concerned provision of items, goods, services, sponsorship, and hospitality to health professionals (§18–§19). There were 102 (5.2%) §2 rulings—i.e., rulings reserved for particularly serious cases of misconduct—and 38 (1.9%) breaches of undertaking (§25).

**Table 3:** UK rulings of breach of selected clauses 2004–2012.

§	Description	Abbreviated Specification	**All Breaches *n*** = **1,950** ^a^	**All Cases *n*** = **597** ^**b**^	**Serious Cases *n*** = **100** ^c^
2	Discredit and confidence	Promotion that “brings discredit to, and reduction of confidence in, the industry.”	102 (5.2%)	92 (15%)	92 (92%)
3	Marketing authorization	Promotion prior to the grant of a marketing authorization (§3.1; *n* = 27), or inconsistent with the terms of marketing authorization and with the particulars listed in the SPC (§3.2; *n* = 86).	113 (5.8%)	88 (15%)	20 (20%)
7	Information, claims, and comparisons	E.g., any information, claim, or comparison must be accurate, be an up-to-date evaluation of all the evidence, and must not mislead (§7.2; *n* = 558); must be capable of substantiation (§7.4; *n* = 193); Promotion must encourage rational medicine use by presenting it objectively (§7.10; *n* = 95).	1,021 (52%)	280 (47%)	28 (28%)
15	Representatives	E.g., representatives must at all times maintain a high standard of conduct and comply with all relevant requirements of the code (§15.2; *n* = 54). Companies must prepare detailed briefing material for medical representatives on the technical aspects of each medicine which they promote. Material must not advocate, either directly or indirectly, any course of action that would be likely to lead to a breach (§15.9; *n* = 27).	96 (4.9%)	81 (14%)	15 (15%)
18	Items for patients; promotional aids; goods and services; agreements to benefit patients	E.g., no gift, pecuniary advantage, or benefit may be supplied, offered, or promised to members of the health professions or to administrative staff in connection with the promotion of medicines or as an inducement to prescribe, supply, administer, recommend, buy, or sell any medicine (§18.1; *n* = 26).	37 (1.9%)	35 (5.9%)	15 (15%)
19	Meetings, hospitality, and sponsorship	E.g., companies must not provide hospitality to members of the health professions and administrative staff except in association with scientific or promotional meetings, congresses, and training (§19.1; *n* = 35).	36 (1.8%)	30 (5.0%)	17 (17%)
22	Relations with the public and the media	E.g. prescription medicines must not be advertised to the public (§22.1; *n* = 31); Information about prescription medicines to the public must be factual and not raise unfounded hopes or be misleading with respect to safety (§22.2; *n* = 53).	84 (4.3%)	55 (9.2%)	13 (13%)
25	Compliance with undertakings	When an undertaking has been given in relation to a ruling, the company concerned must ensure that it complies with that undertaking.	38 (1.9%)	33 (5.5%)	25 (25%)

^a^Percentages indicate cell portion of total breaches. E.g., 5.2% of breaches pertained to §2.

^b^Percentages indicate cell portion of total cases. E.g., 15% of cases involved one or more §2 ruling.

^c^Percentages indicate cell portion of total serious violation cases. E.g., 92% of serious violation cases involved one or more §2 ruling.

In Sweden, over the same period, there were 972 rulings of breach of specific clauses (Table 4 for selected clauses; S2 Table for complete list). Just as for the UK, breaches regarding information, claims, and comparisons were most common, either concerning marketing aimed at health professionals (§4, 26%, *n* = 250), or at the public (§104, 7.5%, *n* = 73). In addition, 16% (*n* = 158) concerned violations of §8 or §10–§12, mandating appropriate documentation and references for claims, as well as fair comparisons. Moreover, as in the UK, a significant number of breaches (§2; 16%, *n* = 156, and §102; 10%, *n* = 100) concerned promotion prior to or inconsistent with the terms of the marketing authorization or the SPC, and 10% (*n* = 100) concerned promotion of prescription drugs to the public (§102). There were 54 breaches (5.6%) of §32–§42, which lay down rules on provision of items, goods, services, sponsorship, and hospitality to health professionals. The Swedish Code does not contain specific clauses on serious violations or failures of undertaking, thereby impeding comparisons among such clauses.

**Table 4:** Sweden rulings of breach of selected clauses 2004–2012.

§	Description	Abbreviated Specification	**All Breaches *n*** = **972** ^a^	**All Cases *n*** = **536** ^b^	**Serious Cases *n*** = **101** ^**c**^
2	Marketing authorization	The SPC constitutes the factual basis for information. Information may only refer to drugs that have received marketing approval.	156 (16%)	156 (29%)	50 (49%)
4	Information, claims, and comparisons	Information must be truthful and may not contain any presentation that directly or indirectly is intended to mislead. E.g., exaggerated claims about properties or effects may not be made (§4.3).	250 (26%)	250 (47%)	67 (66%)
8	Documentation and references	Information as to the quality and efficacy of a drug shall be capable of substantiation by means of documentation of a high scientific standard.	14 (1.4%)	14 (2.6%)	4 (4.0%)
10	Documentation and references	Information that contains quotations, data, etc., from a scientific study or deals with a comparison between drugs based on such a study, must clearly contain information about relevant sources and references.	18 (1.9%)	18 (3.4%)	4 (4.0%)
11	Documentation and references	E.g., a study may not be cited in such a way that it could convey an incorrect or misleading impression of its nature, scope, implementation, or importance (§11.2); Statements of comparisons should be expressed in such a way as to make clearly evident their statistical validity (§11.4).	62 (6.4%)	62 (12%)	22 (22%)
12	Comparisons	Comparisons between effects, active ingredients, costs of treatment, etc., must be presented in such a way that the comparison as a whole is fair.	64 (6.6%)	64 (12%)	22 (22%)
102	Marketing to the public: marketing authorization	Information to the public on prescription drugs shall be supplied only to the extent permitted in the MPA’s provisions and that which applies according to laws and regulations. Information shall be consistent with the SPC: see §2.	100 (10%)	100 (19%)	18 (18%)
104	Marketing to the public: information, claims, comparisons	Information or claim must be truthful and may not contain any presentation in words or pictures that directly or indirectly is intended to mislead. See §4.	73 (7.5%)	73 (14%)	14 (14%)
32–42	Agreement on relations with health care sector	E.g., rules on provision of items, goods, services, sponsorship, and hospitality; Sponsorship of activities/conferences within the operations of the public health care or pharmacy companies may not take place (§38); No gifts or financial benefits may be supplied, offered, or promised to personnel/pharmacy as an incentive to recommend, prescribe, purchase, supply, sell, or administer drugs (§40).	54 (5.6%)	54 (10%)	0

^a^Percentages indicate cell portion of total breaches. E.g., 16% of breaches pertained to §2.

^b^Percentages indicate cell portion of total cases. E.g., 29% of cases involved one or more §2 ruling.

^c^Percentages indicate cell portion of total serious violation cases. E.g., 49% of serious violation cases involved one or more §2 ruling.

### Sanctions for Code Breaches

**Fig 4:**
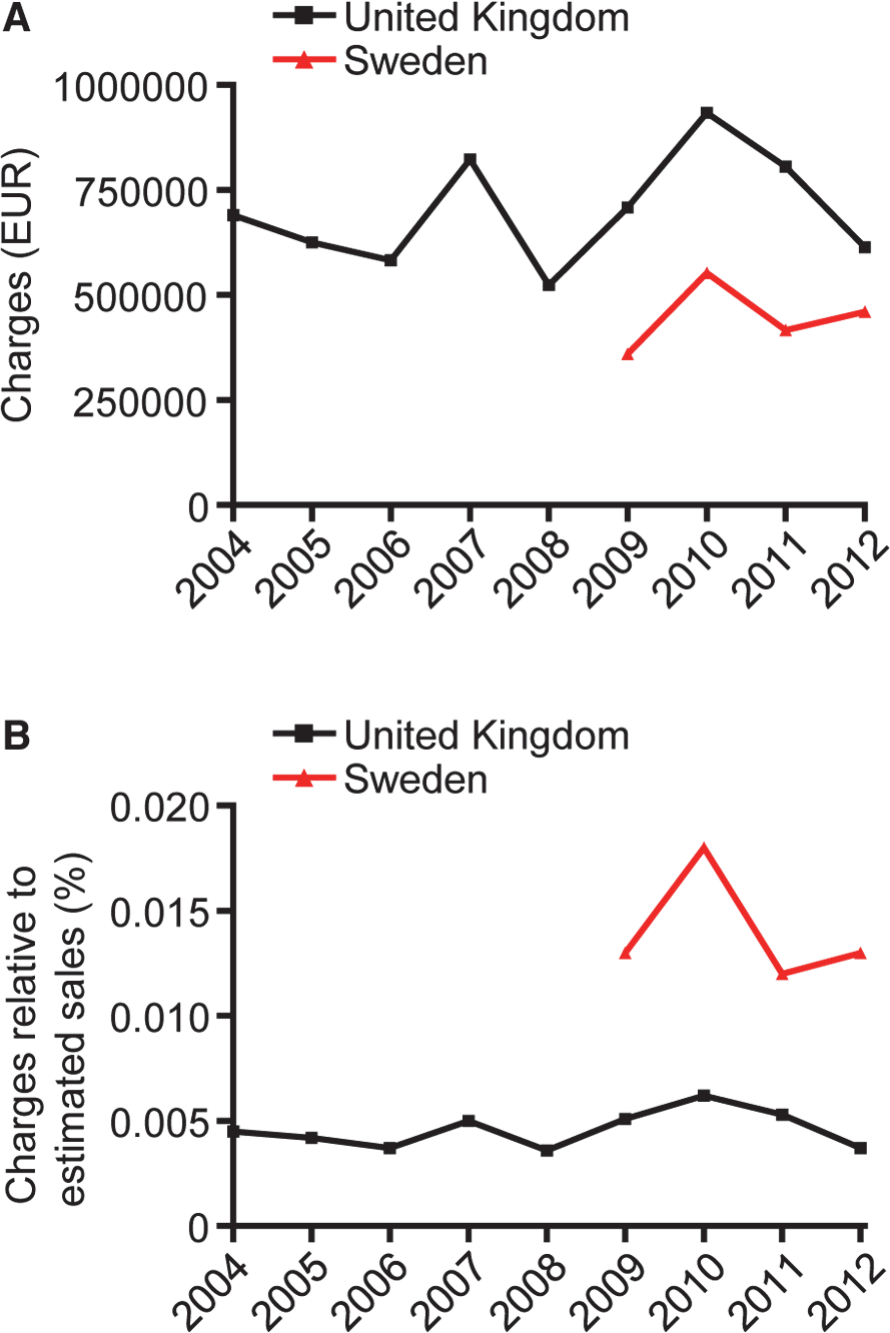
Charges levied on companies by self-regulatory bodies in the United Kingdom and Sweden 2004–2012. Charges in absolute amounts (A) and as percentage of estimated sales at manufacturers’ prices (B) for prescription medicines in the UK and prescription and OTC medicines in Sweden, respectively. Data for Sweden available since 2009.

Fig. 4 shows the economic sanctions imposed on the industry both in absolute numbers and in relation to estimated revenues from drug sales. In Sweden, between 2009 and 2012 (data only available since 2009) charges corresponded to an average of €447,000 per year, or 0.014% of estimated annual revenues. In the UK, sanctions between 2004 and 2012 and between 2009 and 2012 corresponded to an average of €701,000 and €765,000 annually, or 0.0046% and 0.0051% of estimated annual revenues, respectively.

There were no examples of non-economic sanctions in Sweden other than the standard publication of case reports. In the UK, there were 92 cases with one or more §2 ruling for a total of 102 §2 rulings (Table 3), i.e., violations highlighted as particularly serious by self-regulatory bodies, and which are always publicized in the form of advertisements appearing in the professional press. In addition, 19 §2 rulings were associated with one or more of the following non-economic sanctions: audit of the company (*n* = 14), public reprimand (*n* = 9), corrective statement (*n* = 2), recovery of promotional items (*n* = 1), or cash payment (*n* = 1), or temporary suspension from the ABPI (*n* = 3). Eight rulings did not involve breach of §2, but were associated with one or more of the following: audit of the company (*n* = 6), public reprimand (*n* = 2), or recovery of promotional items (*n* = 3). In summary, 110 non-economic sanctions (other than publication) were levied in the UK.

### Serious Violations of Industry Code

**Fig 5:**
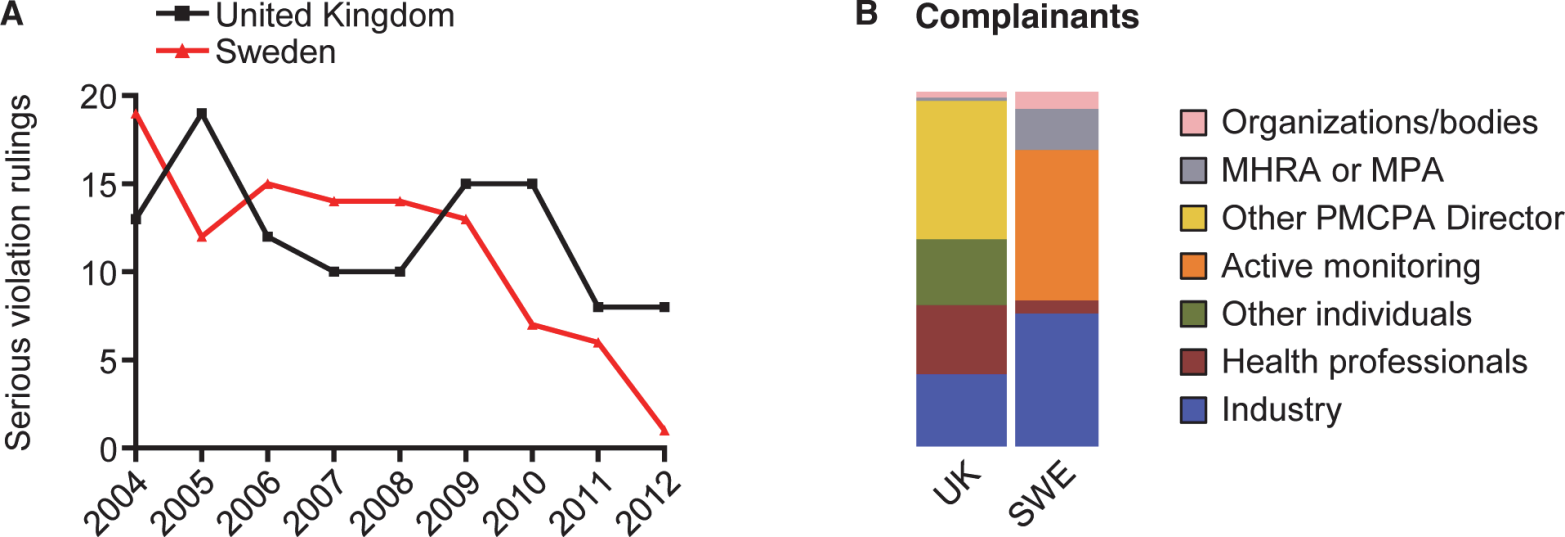
Serious violations in the United Kingdom and Sweden 2004–2012: rulings of breach and complainants. (A) Serious violation rulings per year in the UK (total *n* = 110) and Sweden (total *n* = 101). (B) shows mosaic plot of the source of the complaint for serious violation rulings in each country over the entire period. A few cases had multiple complainants.

In the UK, 100 cases (17% of total) for a total of 110 rulings were thus considered particularly serious by self-regulatory bodies (Table 3; see also Fig. 2). In Sweden the figure was 101 cases (19% of total) (Table 4). There were numerically fewer serious violation rulings in the UK and Sweden in more recent years (Fig. 5A). In Sweden, 44% (*n* = 44) of serious violation cases were initiated following active monitoring (Fig. 5B). In the UK, 40% (*n* = 44) of successful complaints were nominally attributed to the PMCPA Director, but none of these resulted from active monitoring. Only 3.9% (*n* = 4) of cases in Sweden resulted from complaints by health professionals and none from complaints by other individuals, while in the UK the figures were 20% (*n* = 22) and 19% (*n* = 21), respectively (Fig. 5B). Also consistent with differences between the countries we found that, while numerous UK cases pertained to misconduct of representatives (§15) and/or provision of items, goods, services, sponsorship, and hospitality to health professionals (§18), no serious violation rulings in Sweden cited a breach of §32–§42 covering comparable activities (Tables 3–4 for selected clauses; S1–S2 Tables for complete list). Instead, Swedish cases most frequently focused on violation of clauses mandating non-misleading information, claims, and comparisons (i.e., §4, §8, §10–§12, §104).

Forty-six companies were ruled in breach for a serious offence at least once in both countries combined (*n* = 36 in the UK; *n* = 27 in Sweden), and seven companies were ruled in breach for a serious offence more than ten times: Pfizer (*n* = 19; 9.0%), Bayer (*n* = 16; 7.6%), GlaxoSmithKline (*n* = 13; 6.1%), Novo Nordisk (*n* = 12; 5.7%), Novartis (*n* = 12; 5.7%), AstraZeneca (*n* = 11; 5.2%), and Eli Lilly (*n* = 11; 5.2%) (S3 Table). In 2012, four of these companies ranked among the ten largest in terms of global drug sales, and all ranked among the 17 largest [29].

The drug class associated with the highest number of rulings for serious violations in each country were diabetes drugs (UK, *n* = 15; Sweden, *n* = 6; 10% of serious violations) and urologics (Sweden, *n* = 13; UK, *n* = 6; 9%) (S4 Table; see also Fig. 2). A description of each case can be found in S5–S8 Tables. As summarized in Table 5, these 40 rulings revealed one or more violating activities of various types, including—but not limited to—misleading claims (*n* = 23; 58%), failures to comply with undertakings (*n* = 9; 23%), pre-licensing (*n* = 7; 18%) or off-label promotion (*n* = 2; 5%), and promotion of prescription drugs to the public (*n* = 6; 15%).

**Table 5:** Type and frequency of serious violation rulings: diabetes drugs and urologics.

Type of Violation	**UK *n*** = **21**	**SWE *n*** = **19**	Example
Misleading claim	8 (38%)	15 (79%)	Advertisement claimed: “There are no long-term cardio-vascular concerns regarding the use of Actos” (pioglitazone; Takeda). Failed to mention that drug might exacerbate and precipitate heart failure and was contraindicated in patients with, or with a history of, heart failure. (UK 2125/5/08^a^)
Breach of undertaking	3 (14%)	6 (32%)	Bayer marketed Levitra (vardenafil) with claims of rapid onset inconsistent with the SPC despite three successive rulings on this matter. (SWE W950/07; W955/07)
Pre-licensing promotion	6 (29%)	1 (5.3%)	Four §2 rulings regarding pre-licensing promotion of Victoza (liraglutide; Novo Nordisk): on website; via sponsored meetings disguised as scientific and medical meetings; via paid-for insert in medical journal disguised as independent supplement; at diabetes meeting by professor who failed to disclose financial relationship with company. (UK 2234/5/09)
Promotion to the public	5 (24%)	1 (5.3%)	Novo Nordisk promoted Victoza in a newspaper supplement (The Times) on World Diabetes day and, further, did so prior to the granting of marketing authorization. (UK 2202/1/09)
Hospitality	3 (14%)	0	Bayer invited health professionals to a one and a quarter hours meeting on “The medical and surgical management of erectile dysfunction” followed by a champagne reception and gourmet dinner. Hospitality considered “excessive”. (UK 1741/7/05)
Disguised promotion	3 (14%)	0	Article in *Future Prescriber* constituted disguised and pre-licensing promotion of linagliptin by Boehringer-Ingelheim. The article incorrectly claimed that the drug had received marketing authorization, and that it was “safer to use” together with some medications than saxagliptin despite the lack of head-to-head trials. (UK 2424/8/11; 2425/8/11)
Conduct of representative	2 (9.5%)	0	Without apparent company approval, a representative implied that continued funding of an educational post within the local diabetes network could be in danger if the hospital did not increase its use of Lilly insulins. (UK 2044/9/07)
Off-label promotion	1 (4.8%)	1 (5.3%)	Claim in mailing and advertisements, that Glucobay (acarbose, Bayer) had “cardioprotective effect” on patients with IGT and type 2 diabetes constituted off-label promotion since the drug was approved neither for patients with IGT nor for any cardioprotective use. (SWE W647/04; W648/04)
Rules on information	0	1 (5.3%)	Astellas sent letter entitled “Important information for people who work in health care” to doctors. The label “Important information” is only allowed for new information on ADRs, contraindications, restrictions, or withdrawals. The letter contained no such information, but instead promoted Vesicare (solifenacine). The MPA alleged disguised promotion, but the NBL rejected the allegation on this point. (SWE 913/11)

^a^Case number ID: see S5–S8 Tables for additional information.

ADR, adverse drug reaction; IGT, impaired glucose tolerance.

## Discussion

### Frequent and Serious Violations of the Industry Code of Conduct

Our study reveals a substantial number of violations of the two industry codes. Between 2004 and 2012, self-regulatory bodies in Sweden and the UK ruled a total of 536 and 597 cases to be in breach, respectively, which translates to an average of more than one case per week in each country. The Swedish MPA, writing in one of its regular information letters [30], reported similar numbers over a decade ago: “Overall, the number of marketing activities ruled in breach by the NBL and LIF Information Examiner (IGM) amount to some fifty/year, which is about one/week. This is remarkable, given that the drug industry has authorized its own regulatory system, and shows that a critical stance should always be taken regarding pharmaceutical industry marketing practices.”

Nonetheless, data from self-regulatory bodies show fewer complaints and cases ruled in breach over the studied period. However, this is not true for individual matters in breach in the UK, obscuring any simple interpretation of trends. Moreover, there may be numerous reasons for downward trends; for instance, while they may reflect increased compliance, they may also be due to a lower tendency to report breaches. Yet another hypothesis is that the decline reflects a reduction in the total number of marketing campaigns and/or changes in the type of promotion. Consistent with the latter hypothesis, data from Sweden show a 41% decrease in submitted statutory copies over the study period compared to a 23% decrease in violation rulings. US data showing a decline in promotional spending since 2004 also support this hypothesis [31,32]. However, the hypothesis of declining and/or changing marketing activity awaits statistical testing, which requires larger sample sizes.

At this juncture, it is important to point out that for reasons outlined below, the numbers reported herein likely underestimate the unethical conduct of companies. First, UK data do not include activities pertaining to the promotion of OTC drugs. Second, it is implausible that every violation is brought to the attention of self-regulatory bodies [20]. In particular, Code breaches by industry representatives may be among the hardest to document, but also potentially the most serious [33,34]. Third, it may be difficult to retrospectively demonstrate improper conduct, for example to confirm that an industry representative actually said what the complainant alleged. Fourth, there may be lax oversight from self-regulatory bodies, as was suggested in a previous study [20]. Fifth, a number of complaints never progress to become established cases, either because the offence is considered minor and/or because the company promises to immediately rectify the misdemeanor. Sixth, inter-company dialogue allows companies to stop engaging in violative activities without being reprimanded by self-regulatory bodies (which also means that no case report will exist and that activities most likely remain publically unknown). Seventh, if the Codes are weak it may be possible for companies to be in perfect compliance but still disseminate misleading information, as was pointed out previously [35]. Eight, some cases of illicit promotion are handled by national medicines regulatory bodies, rather than by self-regulatory bodies.

This latter point may be particularly relevant in the UK. Both the MPA and the MHRA say they routinely scrutinize promotional material for potential breaches of advertising legislation, and will also consider complaints from all sources, although both strongly encourage the use of the self-regulatory system for complaints involving companies that have ratified the industry Code [11,36]. However, whereas the MPA has delegated responsibility to self-regulatory bodies to the extent that the MPA will itself submit complaints to the NBL, and will typically respect decisions even if they are contrary to the agency’s own view (see the Vesicare example in Table 5), the MHRA at least seems to retain a few more functions by comparison. Thus the MRHA only submits cases to the PMCPA in situations where an initial investigation has found no breach of legislation, but a potential breach of the industry Code [36]. On the other hand, the MHRA will routinely decline to investigate potential breaches of advertising legislation that are under investigation by the PMCPA for potential code breaches, in this way delegating the responsibility to the self-regulatory system [36]. Still, each year a number of potential breaches of advertising legislation are investigated by the MHRA. Some of these involve companies that have ratified the industry Code [37].

This study also reveals that self-regulatory bodies also considered about 20% of cases ruled in breach to be particularly serious, equating to an average of about one case per month in each country. In both countries, there were numerically fewer rulings of serious violations towards the end of the studied period. However, we note that the number of serious violation rulings increased again in 2013 compared to 2012—in Sweden from one to nine; in the UK from eight to 15—suggesting a need to analyze longer time series. As shown by our analysis of cases involving promotion of diabetes drugs or urologics, such serious violations reveal conduct that may compromise patient safety either directly by biasing prescribing practices (e.g., misleading claims [38]), or indirectly by undermining the regulatory system for drug marketing approvals (e.g., pre-licensing and off-label promotion [39]). Importantly, serious violations are not restricted to just a few products or companies. Thus between 2004 and 2012, 46 existing companies were ruled in breach for a serious offence at least once in the UK and Sweden combined, and many were repeat offenders including some of the largest pharmaceutical companies in the world. The prevalence and severity of breaches testifies to a discrepancy between the ethical standard approved by companies and codified in industry codes of conduct and the actual conduct of the industry.

### Patterns in Industry Deviance

Self-regulatory bodies in Sweden and the UK ruled a total of 972 and 1,950 breaches to individual clauses, respectively, which translates into an average of almost two (Sweden) and more than four (UK) per week. Note, however, that a head-to-head comparison between the two countries is not possible because in the UK, but not in Sweden, each case is subdivided into multiple matters that are ruled upon independently, which compounds the number of breaches. By far, the most common reported violation in both countries relates to misleading product information, claims, or comparisons, which is not surprising given that a number of studies investigating the quality of claims made in medical journal advertisements have reached the conclusion that such claims are often misleading [9,20,40–47].

In light of the silence in Europe compared with the US concerning off-label promotion, it is perhaps more surprising that numerous breaches in both study countries concerned pre-licensing promotion or promotion inconsistent with the terms of the marketing authorization or SPC. In the UK, for instance, such promotion accounted for 5.8% of breaches, while 20% of rulings of serious violation cited the corresponding article (i.e., §3). However, even after excluding cases of pre-licensing promotion, accounting for about one-third of violations judging from the UK data where such a distinction is made, it cannot be assumed that the remaining violations represent cases of off-label promotion. The reason is that the SPC includes additional information aside from the authorized uses of the drug, such as clinical trial data and pharmacological properties. Nevertheless, our qualitative analysis of promotion of diabetes drugs and urologics did reveal examples of off-label promotion in both countries (see also [48]).

Another notable piece of information is that 4.3% of breaches in the UK and 10% of breaches in Sweden apparently concerned promotion of prescription drugs to the public. This type of promotion is in conflict not only with EU law [49], but also with the current position of the European industry trade group, which is that it does “not advocate U.S.-style direct-to-consumer advertising as an appropriate model for Europe” (p. 822) [50]. This situation again points to a discrepancy between the official discourse of the industry and actual practice.

Thus far this discussion has highlighted patterns of industry deviance shared by Sweden and the UK. However, the pattern of Code breaches for particularly serious cases also highlighted discrepancies, for example in regard to the conduct of representatives and the provision of items, goods, services, sponsorship, and hospitality to health professionals. It remains to be determined whether such discrepancies are due to differences in industry marketing strategies between the countries, or if they (also) reflect cross-country differences that impact the tendency of actors to report breaches, such as demographic (population size differences, currently 63.7 million versus 9.6 million, or differences in the number of health professionals), cultural (e.g., trust in regulators or industry), or procedural differences (e.g., names of complainants are kept confidential in the UK). Interestingly, in support of the latter explanation, we found a number of complaints from industry employees and ex-employees in the UK, but not in Sweden.

### Creating Incentives for Industry

We interpret the arguably high rate of code violations as evidence that self-regulation has failed to sufficiently deter industry from engaging in frequent and sometimes serious unethical practices, as judged by the industry’s own standards. The lack of sufficient deterrent effect is also supported by our findings that many companies were repeat offenders and that companies on numerous occasions ignored rulings as evidenced by subsequent rulings of failures to comply with undertakings (see Levitra example in Table 5 for a particularly illustrative case). One reason for this high rate could be that costs for violations are too low. Consistent with this, we found that charges incurred amounted to an average of about 0.014% and 0.005% of estimated yearly revenues from drug sales in Sweden and the UK, respectively. To put this into context, consider that industry promotion in the US in 2010 was estimated at 9% of sales revenue [31], though the real figure is likely to be significantly higher [51], with the WHO citing a figure of about 30% globally [52]. Notably, the charges incurred on violating companies in the UK and Sweden are considerably below the level experienced by the industry in the US. Thus from January 2009 through September 2012 the US Department of Justice recovered nearly US$10.5 billion in whistleblower suits under the False Claims Act [53], corresponding to 0.8% of total US prescription drug sales 2009–2012 [54]. Some have argued that the US penalties threaten to cripple the industry financially [55]. Others have maintained that even the US fines are too low as evidenced by the lack of long-term impact on company stock prices and the profitable nature of illicit activities [56, 57].

In fairness, charges in the UK and Sweden are not designed to damage corporations financially; presently, they reflect the cost of administering the self-regulatory system—hence the term “administrative charges” used in the UK. In fact, because charges in the UK are not even sufficient to cover the costs of managing the self-regulatory system, including rent to the ABPI, corporations also pay an annual levy, in 2012 about €2,200–€17,400 depending on the size of the company [58]. In Sweden, LIF may similarly inject extra capital to cover expenses, but the amounts remain undisclosed. Arguably, the fact that infringing corporations pay charges aimed at keeping the self-regulatory system afloat, rather than providing compensation for damages caused by—and profits generated as a result of—illicit promotion represents a major weakness in both systems.

Ultimately however, economic penalties may not be enough to deter companies from illicit promotion [3]. Tarnishing the reputation of violating companies might be equally important, if this has major negative effect on business performance. According to the PMCPA, “Publicity is the main sanction when breaches of the Code are ruled” [59] and as a matter of policy, when a breach of §2 is ruled, a public reprimand is issued. From this perspective a major weakness of the Swedish system is the lack of an equivalent to the UK §2 and a policy to publicly reprimand companies. As a matter of fact, despite a provision that allows the IGM and NBL to require companies to issue corrective statements, evidently this has never happened in Sweden, despite frequent and serious violations of the Code.

### Active Monitoring and Pre-vetting: Important Regulatory Instruments

One of the most remarkable findings of this study is that despite the fact that active monitoring is supposedly a major function of the PMCPA under the Code, and despite a memorandum involving the ABPI, PMCPA, and MHRA that since 2005 tasks the PMCPA with routine scrutiny of promotional material [36], there is little *prima facie* evidence that the authority fulfills its duties in this respect to any significant extent. This finding is particularly problematic in light of data from Sweden suggesting that active monitoring is an important mechanism for exposing violations, including particularly serious ones. A key feature of the Swedish system is that companies are required to submit statutory copies of drug promotion. Although limited to print material, mailings, Internet ads, and films, this requirement may nonetheless facilitate identification of material in breach.

The fact that active monitoring seems viable in Sweden points to another issue worth pondering: if the IGM is able to scrutinize material after publication, there is no apparent reason why it could not vet material beforehand, were it to acquire additional resources. Notably, the IGM already pre-vets vaccination programs and drug information on company websites aimed at the public. The PMCPA, by contrast, does not pre-vet material, but the Proprietary Association of Great Britain (PAGB) pre-vets consumer advertisements for OTC drugs [60]. Moreover, the MHRA has pre-vetted advertisements for new products, products affected by safety concerns, or major new indications since 2005 after the House of Commons Select Committee issued a recommendation in its landmark report on the undue influence of the industry [61]. The Swedish MPA, by contrast, does not pre-vet material, which would likely be in conflict with the prohibition of censorship in the Swedish constitution.

However, despite assurances from the MHRA that “the vetting procedure is successful in improving standards of advertising” (p. 14) [60], the UK medicines authority recently announced that it was preparing to relax vetting efforts [12]. According to the MHRA’s seventh annual report on advertising [60], publicity materials for 40 products were vetted in 2012, but from now on, instead of assessing many advertising pieces, the MHRA announced it would be looking at “perhaps a small number of key pieces, and that would be done in a very light touch way” [12]. Yet, rather than supporting a light touch approach to vetting, the abundance of misleading claims despite pre-vetting efforts would seem to mandate an intensified effort instead.

### Policy Recommendations

As argued here, reforms that may improve the quality of medicines information include intensified pre-vetting and active monitoring efforts in conjunction with fines that effectively deter industry from illicit promotion, as well as greater publicity following rulings. This list is not exhaustive (for example, see [62] for additional suggestions). Such reforms could have relevance for other countries as well [19] although more analyses of promotion regulatory systems are needed to substantiate this contention. Tables 1 and 2 present additional features of regulatory systems worth considering in future analyses, such as the extent of financial disclosure, rules regarding the complaints procedure, and type of information made available for public scrutiny.

Yet, despite the importance of improving current regulatory arrangements in an effort to ensure unbiased medicines information, such initiatives alone are insufficient to achieve this goal. The reason relates to the additional layers of industry bias that cannot be addressed by simply improving oversight and increasing penalties, such as practices related to selection, design, and publication of clinical studies [63–67]. From this perspective, one attractive model to counterbalance the influence of industry was implemented by the Italian medicines authorities, requiring companies to contribute 5% of yearly expenditures devoted to promotional initiatives aimed at physicians to a fund for independent clinical research [68]. The cost of pharmaceuticals throughout the EU reached more than €218 billion in 2010 [69]. After correcting for pharmacies’ margins (−17.4% of expenses using to the Swedish example), and assuming that industry spends 10%–30% (see above) of revenues on marketing, this would translate into €0.9–€2.7 billion in tax revenues in the EU. To put this into perspective, the entire 2013 supranational EU budget for research and innovation amounted to €10.8 billion.

Another policy innovation worth considering is the creation of industry-independent organizations in each country dedicated to offering information on treatments, the funding for which would be collected as a fixed percentage of charges levied by regulatory bodies. Crucially, because the budget for this organization would be proportional to rule breaking by industry (assuming discovery), this financial arrangement would not only have the advantage of incentivizing industry to follow the rules, but would also incentivize the industry-independent organizations to act as a watchdog against violative industry conduct.

### Limitation

This study has several limitations. First, although the UK and Swedish systems share a number of relevant features facilitating direct comparison, including procedural similarities, there are important differences that compound the analysis, including differences in market size and the fact that the Swedish but not UK data include breaches pertaining to promotion of OTC medicines. Second, calculations were based on data provided by self-regulatory bodies with no possibility to independently check for accuracy. Third, as noted above, the numbers presented herein most likely underestimate the unethical conduct of the industry. Fourth, we did not assess the frequency of violative versus non-violative conduct. For these reasons, the debate will surely continue with critics of industry promotion contending that the cases identified are merely the “tip of the iceberg” of misconduct, while defenders of industry promotion will contend that the critique suffers from the “denominator neglect” discussed by Stossel and Stell [55] in relation to alleged dangers of industry bias in academia—that the many cases of proper conduct dwarf the comparatively few cases that populate the numerator. Yet, as this study has shown, the numerator of industry misconduct is anything but negligible.

### Conclusion

The prevalence and severity of breaches identified by self-regulatory bodies in the UK and Sweden testifies to a discrepancy between the ethical standard codified in industry codes of conduct and the actual conduct of the industry. Policies that might improve the quality of medicines information include intensified pre-vetting and active monitoring efforts in conjunction with larger fines, as well as greater publicity following rulings. Future studies need to assess the effectiveness of such policies and investigate promotion and its regulation in other countries.

## Supporting Information

S1 Fig Statutory copies of drug promotion sent to the IGM by companies in Sweden 1998–2012 by type. (TIF)

S1 Table UK rulings of breach 2004–2012: complete list of clauses. (PDF)

S2 Table Sweden rulings of breach 2004–2012: complete list of clauses. (PDF)

S3 Table Serious violation rulings in the UK and Sweden 2004–2012: companies. (PDF)

S4 Table Serious violation rulings in the UK and Sweden 2004–2012: promoted drugs. (PDF)

S5 Table Serious violations: promotion of diabetes drugs in the UK. (PDF)

S6 Table Serious violations: promotion of diabetes drugs in Sweden. (PDF)

S7 Table Serious violations: promotion of urologics in the UK. (PDF)

S8 Table Serious violations: promotion of urologics in Sweden. (PDF)

S1 Text Additional information on the qualitative coding and analysis process. (DOCX)

## Abbreviations

ABPI: Association of the British Pharmaceutical Industry
ATC: Anatomic Therapeutic Chemical; EU, European Union
IGM: Informationsgranskningsmannen (Pharmaceutical Industry’s Information Examiner)
LIF: Läkemedelsindustriföreningen (the trade association for the research-based pharmaceutical industry in Sweden)
MHRA: Medicines and Healthcare Products Regulatory Agency (the UK medicines regulatory authority)
MPA: Medical Products Agency (the Swedish medicines regulatory authority)
NBL: Nämnden för Bedömning av Läkemedelsinformation (Information Practices Committee)
OTC: over-the-counter
PMCPA: Prescription Medicines Code of Practice Authority
SPC: summary of product characteristics
