# Supplementary figures and images for: Complaints, Complainants, and Rulings Regarding Drug Promotion in the United Kingdom and Sweden 2004–2012: A Quantitative and Qualitative Study of Pharmaceutical Industry Self-Regulation
Source: PLoS Med. 2015 Feb 17;12(2):e1001785. doi: 10.1371/journal.pmed.1001785 (PMC4331559; doi:10.1371/journal.pmed.1001785)

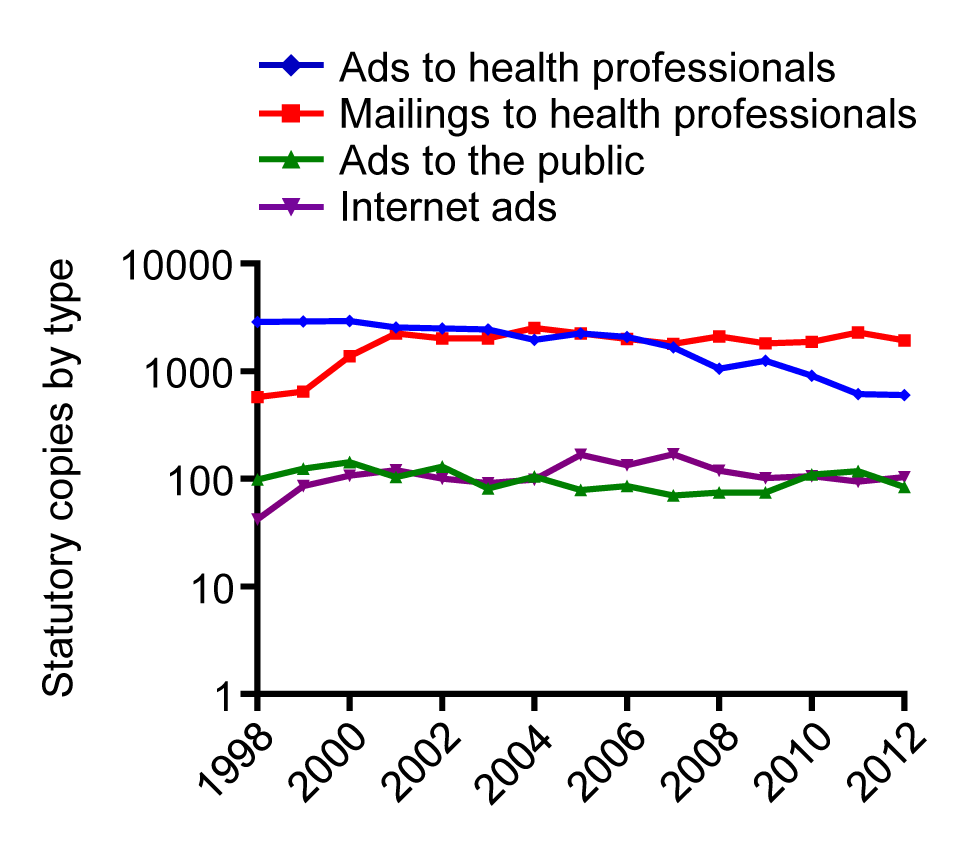

Supplement: S1 Fig — (TIF) [file pmed.1001785.s001.tif]
