# Supplementary material for: Complaints, Complainants, and Rulings Regarding Drug Promotion in the United Kingdom and Sweden 2004–2012: A Quantitative and Qualitative Study of Pharmaceutical Industry Self-Regulation
Source: PLoS Med. 2015 Feb 17;12(2):e1001785. doi: 10.1371/journal.pmed.1001785 (PMC4331559; doi:10.1371/journal.pmed.1001785)
Supplement: S1 Table — (PDF) [file pmed.1001785.s002.pdf]

**Table S1. UK rulings of breach 2004-2012: complete list of clauses**

| § | Description | All breaches n=1950[a] | All cases n=597[b] | Serious cases n=100[c] |
|---|---|---|---|---|
| 1 | Scope of code and definitions | 5 (0.3%) | 5 (0.8%) | 3 (3.0%) |
| 2 | Discredit and confidence | 102 (5.2%) | 92 (15%) | 92 (92%) |
| 3 | Marketing authorization | 113 (5.8%) | 88 (15%) | 20 (20%) |
| 4 | Prescribing information and other obligatory information | 64 (3.3%) | 45 (7.5%) | 9 (9.0%) |
| 5 | Abbreviated advertisements | 1 (0.1%) | 1 (0.2%) | 0 |
| 6 | Journal advertising | 2 (0.1%) | 2 (0.3%) | 0 |
| 7 | Information, claims and comparisons | 1021 (52%) | 280 (47%) | 28 (28%) |
| 8 | Disparaging references | 33 (1.7%) | 27 (4.5%) | 5 (5.0%) |
| 9 | High standards; format; suitability and causing offence; sponsorship | 249 (13%) | 210 (35%) | 77 (77%) |
| 10 | Reprints and quotations | 3 (0.2%) | 3 (0.5%) | 0 |
| 11 | Distribution of promotional material | 3 (0.2%) | 2 (0.3%) | 0 |
| 12 | Disguised promotion | 32 (1.6%) | 25 (4.2%) | 9 (9.0%) |
| 13 | Non-interventional studies | 0 | 0 | 0 |
| 14 | Certification | 16 (0.8%) | 13 (2.2%) | 4 (4.0%) |
| 15 | Representatives | 96 (4.9%) | 81 (14%) | 15 (15%) |
| 16 | Training | 5 (0.3%) | 4 (0.7%) | 0 |
| 17 | Provision of medicines and samples | 2 (0.1%) | 2 (0.3%) | 0 |
| 18 | Items for patients; promotional aids; provision of goods and services; agreements to benefit patients | 37 (1.9%) | 35 (5.9%) | 15 (15%) |
| 19 | Meetings, hospitality and sponsorship | 36 (1.8%) | 30 (5.0%) | 17 (17%) |
| 20 | Consultants | 2 (0.1%) | 2 (0.3%) | 2 (2.0%) |
| 21 | Scientific services | 0 | 0 | 0 |
| 22 | Relations with the public and media | 84 (4.3%) | 55 (9.2%) | 13 (13%) |
| 23 | Relations with patient organizations | 3 (0.2%) | 2 (0.3%) | 1 (1.0%) |
| 24 | Internet | 3 (0.2%) | 1 (0.2%) | 0 |
| 25 | Compliance with undertakings | 38 (1.9%) | 33 (5.5%) | 25 (25%) |

[a] Percentages indicate cell portion of total breaches. E.g. 5.2% of breaches pertained to §2.
[b] Percentages indicate cell portion of total cases. E.g. 15% of cases involved one or more §2 ruling.
[c] Percentages indicate cell portion of total serious violation cases. E.g. 92% of serious violation cases involved one or more §2 ruling.
