# Supplementary material for: Complaints, Complainants, and Rulings Regarding Drug Promotion in the United Kingdom and Sweden 2004–2012: A Quantitative and Qualitative Study of Pharmaceutical Industry Self-Regulation
Source: PLoS Med. 2015 Feb 17;12(2):e1001785. doi: 10.1371/journal.pmed.1001785 (PMC4331559; doi:10.1371/journal.pmed.1001785)
Supplement: S2 Table — (PDF) [file pmed.1001785.s003.pdf]

Table S2. Sweden rulings of breach 2004-2012: complete list of clauses

§	Description	All breaches n=972[a]	All cases n=536[b]	Serious cases n=101[c]
1	General principles: marketing to health professionals	6 (0.6%)	6 (1.1%)	6 (5.9%)
2	Marketing authorization	156 (16%)	156 (29%)	50 (49%)
3	High standards, good taste and causing offence	4 (0.4%)	4 (0.7%)	1 (1.0%)
4	Information, claims and comparisons	250 (26%)	250 (47%)	67 (66%)
5	Disguised promotion and disclosure	7 (0.7%)	7 (1.3%)	1 (1.0%)
6	Information on publication year or date	2 (0.2%)	2 (0.4%)	1 (1.0%)
7	Information must be up-to-date	2 (0.2%)	2 (0.4%)	1 (1.0%)
8	Documentation and references – of high scientific standards	14 (1.4%)	14 (2.6%)	4 (4.0%)
9	Documentation and references – applicability	2 (0.2%)	2 (0.4%)	1 (1.0%)
10	Documentation and references – sources cited	18 (1.9%)	18 (3.4%)	4 (4.0%)
11	Documentation and references – fair and balanced presentation	62 (6.4%)	62 (12%)	22 (22%)
12	Comparisons	64 (6.6%)	64 (12%)	22 (22%)
13	Disparaging references and discreditation	8 (0.8%)	8 (1.5%)	2 (2.0%)
14	Rules on disseminating promotional information	0	0	0
15	Rules on disseminating important information	1 (0.1%)	1 (0.2%)	1 (1.0%)
16	Rules regarding the form of the information	0	0	0
17	Prescribing information and other obligatory information	37 (3.8%)	37 (6.9%)	6 (5.9%)
18	Prescribing information and other obligatory information	0	0	0
19	Legibility of reproduced SPC or catalogue text	10 (1.0%)	10 (1.9%)	0
20	Format and size of printed material	0	0	0
21	Verbal information – definition and aims	0	0	0
22	Verbal information – conduct of representatives	1 (0.1%)	1 (0.2%)	0
23	Verbal information – timely notification of recipient	2 (0.2%)	2 (0.4%)	0
24	Verbal information – hospitality and compensations	1 (0.1%)	1 (0.2%)	0
25	Verbal information – information on addressees, their right to comment	0	0	0
26	Drug samples	0	0	0
27	Rules on responsibility	2 (0.2%)	2 (0.4%)	0
28	Bearer of responsibility	4 (0.4%)	4 (0.7%)	0
29	Liaison officer	0	0	0
30	Burden of proof	0	0	0
31	Statutory copies	1 (0.1%)	1 (0.2%)	0
32-42	Agreement on relations with healthcare sector	54 (5.6%)	54 (10%)	0
43-56	Rules on non-interventional studies	0	0	0
57	Criteria for providing financial support to National Quality Registers	0	0	0

Table S2. Cont'd: Sweden rulings of breach 2004-2012: complete list of clauses

101	General principles: marketing to the public	4 (0.4%)	4 (0.7%)	1 (1.0%)
102	Marketing to the public: Marketing authorization	100 (10%)	100 (19%)	18 (18%)
103	Marketing to the public: High standards, good taste and causing offence	8 (0.8%)	8 (1.5%)	3 (3.0%)
104	Marketing to the public: Information, claims and comparisons	73 (7.5%)	73 (14%)	14 (14%)
105	Marketing to the public: Disguised promotion and disclosure	17 (1.8%)	17 (3.2%)	2 (2.0%)
106	Marketing to the public: Information on publication year or date	1 (0.1%)	1 (0.2%)	0
107	Marketing to the public: Information must be up-to-date	2 (0.2%)	2 (0.4%)	2 (2.0%)
108	Marketing to the public: Documentation and references (see §8)	6 (0.6%)	6 (1.1%)	0
109	Marketing to the public: Documentation and references (see §9)	0	0	0
110	Marketing to the public: Documentation and references (see §10)	1 (0.1%)	1 (0.2%)	0
111	Marketing to the public: Documentation and references (see §11)	1 (0.1%)	1 (0.2%)	1 (1.0%)
112	Marketing to the public: Comparisons	4 (0.4%)	4 (0.7%)	0
113	Marketing to the public: Disparaging references and discreditation	2 (0.2%)	2 (0.4%)	0
114	Marketing to the public: Rules on disseminating promotional information	0	0	0
115	Marketing to the public: Rules on disseminating important information	1 (0.1%)	1 (0.2%)	0
116	Marketing to the public: Rules regarding the form of the information	0	0	0
117	Marketing to the public: Instructions and obligatory information	39 (4.0%)	39 (7.3%)	0
119	Marketing to the public: Legibility	5 (0.5%)	5 (0.9%)	0
120	Marketing to the public: Format and size of printed material	0	0	0
127	Marketing to the public: Rules on responsibility	0	0	0
128	Marketing to the public: Bearer of responsibility	0	0	0
129	Marketing to the public: Liaison officer	0	0	0
130	Marketing to the public: Burden of proof	0	0	0
131	Marketing to the public: Statutory copies	0	0	0

[a] Percentages indicate cell portion of total breaches. E.g. 16% of breaches pertained to §2.
[b] Percentages indicate cell portion of total cases. E.g. 29% of cases involved one or more §2 ruling.
[c] Percentages indicate cell portion of total serious violation cases. E.g. 49% of serious violation cases involved one or more §2 ruling.
