# Supplementary material for: Complaints, Complainants, and Rulings Regarding Drug Promotion in the United Kingdom and Sweden 2004–2012: A Quantitative and Qualitative Study of Pharmaceutical Industry Self-Regulation
Source: PLoS Med. 2015 Feb 17;12(2):e1001785. doi: 10.1371/journal.pmed.1001785 (PMC4331559; doi:10.1371/journal.pmed.1001785)
Supplement: S3 Table — (PDF) [file pmed.1001785.s004.pdf]

Table S3. Serious violation rulings in the UK and Sweden 2004-2012: companies

Company[a]	UK n=110[b]	SWE n=101	Total n=211
Abbott	4 (3.6%)	4 (4.0%)	8 (3.8%)
Alcon	0	1 (1.0%)	1 (0.5%)
Allergan	7 (6.4%)	0	7 (3.3%)
Amgen	0	3 (3.0%)	3 (1.4%)
ApoPharma	1 (0.9%)	0	1 (0.5%)
Astellas	1 (0.9%)	5 (5.0%)	6 (2.8%)
AstraZeneca	6 (5.5%)	5 (5.0%)	11 (5.2%)
Baxter	0	1 (1.0%)	1 (0.5%)
Bayer	7 (6.4%)	9 (7.9%)	16 (7.6%)
Biogen Idec	0	1 (1.0%)	1 (0.5%)
Boehringer Ingelheim	4 (3.6%)	3 (3.0%)	7 (3.3%)
Bristol-Myers Squibb	1 (0.9%)	0	1 (0.5%)
Chiesi	2 (1.8%)	0	2 (0.9%)
Daiichi Sankyo	3 (2.7%)	0	3 (1.4%)
Eli Lilly	7 (6.4%)	4 (4.0%)	11 (5.2%)
Ferring	1 (0.9%)	1 (1.0%)	2 (0.9%)
GlaxoSmithKline	7 (6.4%)	6 (5.9%)	13 (6.1%)
Grünenthal	3 (2.7%)	0	3 (1.4%)
Janssen-Cilag	2 (1.8%)	3 (3.0%)	5 (2.4%)
Johnson & Johnson	0	4 (4.0%)	4 (1.9%)
Leo Pharma	0	1 (1.0%)	1 (0.5%)
Lundbeck	0	3 (4.0%)	3 (1.4%)
Menarini	1 (0.9%)	0	1 (0.5%)
Merck KGaA	3 (2.7%)	2 (2.0%)	5 (1.9%)
Merck Sharp & Dohme	3 (2.7%)	4 (4.0%)	7 (3.3%)
Merz	2 (1.8%)	0	2 (0.9%)
Mundipharma	0	2 (2.0%)	2 (0.9%)
Napp	2 (1.8%)	0	2 (0.9%)
Novartis	1 (0.9%)	11 (11%)	12 (5.7%)
Novo Nordisk	7 (6.4%)	5 (5.0%)	12 (5.7%)
Orion	1 (0.9%)	0	1 (0.5%)
Otsuka	1 (0.9%)	0	1 (0.5%)
Pfizer	10 (9.0%)	9 (8.9%)	19 (9.0%)
ProStrakan	2 (1.8%)	0	2 (0.9%)
Recip	0	2 (2.0%)	2 (0.9%)
Roche	6 (5.4%)	2 (2.0%)	8 (3.8%)
Sanofi	2 (1.8%)	6 (5.9%)	8 (3.8%)
SBL Vaccin	0	1 (1.0%)	1 (0.5%)
Servier	1 (0.9%)	0	1 (0.5%)
Shire	2 (1.8%)	0	2 (0.9%)
Solvay	1 (0.9%)	0	1 (0.5%)
Takeda	4 (3.6%)	3 (3.0%)	7 (3.3%)

Table S3 cont'd. Serious violation rulings in the UK and Sweden 2004-2012: companies

Teva	3 (2.7%)	0	3 (1.4%)
UCB	1 (0.9%)	0	1 (0.5%)
Vifor	4 (3.6%)	0	4 (1.9%)
Warner Chilcott	3 (2.7%)	0	3 (1.4%)

[a] List based on existing companies. In cases where companies merged with or were acquired by another company subsequent to the violation ruling (e.g. Wyeth by Pfizer; Nycomed by Takeda) we assigned the breach to the existing company (i.e. Pfizer and Takeda, respectively). In cases where the violation was by a subsidiary company (e.g. McNeil) we assigned the breach to the parent company (i.e. Johnson & Johnson).

[b] A few cases involved multiple companies or multiple §2 breaches.
