# Supplementary material for: Complaints, Complainants, and Rulings Regarding Drug Promotion in the United Kingdom and Sweden 2004–2012: A Quantitative and Qualitative Study of Pharmaceutical Industry Self-Regulation
Source: PLoS Med. 2015 Feb 17;12(2):e1001785. doi: 10.1371/journal.pmed.1001785 (PMC4331559; doi:10.1371/journal.pmed.1001785)
Supplement: S4 Table — (PDF) [file pmed.1001785.s005.pdf]

Table S4. Serious violation rulings in the UK and Sweden 2004-2012: promoted drugs

Therapeutic class by ATC Code	UK[a] n=110	SWE n=101	Total n=211
**A02** Drugs for acid related disorders	3 (2.7%)	3 (3.0%)	6 (2.8%)
**A07** Antidiarrheals	1 (0.9%)	0	1 (0.5%)
**A08** Antiobesity preparations	4 (3.6%)	4 (4.0%)	8 (3.8%)
**A10** Drugs used in diabetes	15 (14%)	6 (5.9%)	21 (10%)
**A11** Vitamins	0	1 (1.0%)	1 (0.5%)
**A12** Mineral supplements	1 (0.9%)	0	1 (0.5%)
**A16** Other alim. tract and metabol. products	1 (0.9%)	0	1 (0.5%)
**B01** Antithrombotic agents	4 (3.6%)	1 (1.0%)	5 (2.4%)
**B02** Antihemorrhagics	0	1 (1.0%)	1 (0.5%)
**B03** Antianemic preparations	4 (3.6%)	1 (1.0%)	5 (2.4%)
**C01** Cardiac therapy	0	1 (1.0%)	1 (0.5%)
**C05** Vasoprotectives	1 (0.9%)	0	1 (0.5%)
**C07** Beta blocking agents	1 (0.9%)	0	1 (0.5%)
**C09** Agents act. on renin-angiotensin syst.	3 (2.7%)	6 (5.9%)	9 (4.3%)
**C10** Lipid modifying agent	8 (7.2%)	0	8 (3.8%)
**D03** Prepar. for treatment of wounds/ulcers	0	1 (1.0%)	1 (0.5%)
**D10** Anti-acne preparations	1 (0.9%)	0	1 (0.5%)
**D11** Other dermatological preparations	0	1 (1.0%)	1 (0.5%)
**G03** Sex horm. and modul. of genital syst.	3 (2.7%)	6 (5.9%)	9 (4.3%)
**G04** Urologicals	6 (5.5%)	13 (13%)	19 (9.0%)
**J01** Antibacterials for systemic use	0	1 (1.0%)	1 (0.5%)
**J02** Antimycotics for systemic use	2 (1.8%)	1 (1.0%)	3 (1.4%)
**J06** Immune sera and immunoglobulins	0	1 (1.0%)	1 (0.5%)
**J07** Vaccines	1 (0.9%)	6 (5.9%)	7 (3.3%)
**L01** Antineoplastic agents	4 (3.6%)	5 (5.0%)	9 (4.3%)
**L02** Endocrine therapy	2 (1.8%)	0	2 (0.9%)
**L03** Immunostimulants	2 (1.8%)	1 (1.0%)	3 (1.4%)
**L04** Immunosuppressants	0	7 (6.9%)	7 (3.3%)
**M01** Anti-inflamm. and antirheumatic prod.	3 (2.7%)	6 (5.9%)	9 (4.3%)
**M03** Muscle relaxants	8 (7.2%)	0	8 (3.8%)
**M05** Drugs for treatment of bone diseases	1 (0.9%)	2 (2.0%)	3 (1.4%)
**N01** Anesthetics	2 (1.8%)	1 (1.0%)	3 (1.4%)
**N02** Analgesics	8 (7.2%)	8 (7.9%)	16 (7.6%)
**N03** Antiepileptics	1 (0.9%)	1 (1.0%)	2 (0.9%)
**N04** Anti-parkinson drugs	1 (0.9%)	2 (2.0%)	3 (1.4%)
**N05** Psycholeptics	2 (1.8%)	1 (1.0%)	3 (1.4%)
**N06** Psychoanaleptics	1 (0.9%)	5 (5.0%)	6 (2.8%)
**N07** Other nervous system drugs	4 (3.6%)	4 (4.0%)	8 (3.8%)
**R03** Drugs for obstructive airway diseases	2 (1.8%)	3 (3.0%)	5 (2.4%)
**R05** Cough and cold preparations	1 (0.9%)	1 (1.0%)	2 (0.9%)
**S01** Ophthalmologicals	0	1 (1.0%)	1 (0.5%)
**V03** All other therapeutic products	1 (0.9%)	0	1 (0.5%)
**no drug**	12 (11%)	0	12 (5.7%)

[a] A few UK cases involved multiple drugs
