# Supplementary material for: Complaints, Complainants, and Rulings Regarding Drug Promotion in the United Kingdom and Sweden 2004–2012: A Quantitative and Qualitative Study of Pharmaceutical Industry Self-Regulation
Source: PLoS Med. 2015 Feb 17;12(2):e1001785. doi: 10.1371/journal.pmed.1001785 (PMC4331559; doi:10.1371/journal.pmed.1001785)
Supplement: S5 Table — (PDF) [file pmed.1001785.s006.pdf]

Table S5. Serious violations: promotion of diabetes drugs in the UK

Case no.	Drug	Company	Case summary	Violation
1545/1/04	Avandia (rosiglitazone)	GSK	Without apparent company approval, GSK representative produced and distributed bogus NHS guidelines for the use of thiazolidinediones. The bogus guidelines referred only to rosiglitazone among thiazolidinediones.	Disguised promotion; Conduct of representative
1603/7/04	Levemir (Insulin detemir)	Novo Nordisk	Novo Nordisk arranged for meeting for health professionals at floating restaurant to promote Levemir. The dinner for 95 attendees had cost over £11000 and had started with champagne and canapés and there had been live music and dancers. At another meeting, health professionals were invited to the executive suite of a football venue to watch the match between England and Croatia. Meeting had started with champagne and canapés. According to the invitation, there was a buffet dinner and the bar would be open during the game. Arrangements were found "unacceptable".	Hospitality
1689/3/05	Avandament (rosiglitazone/ metformin)	GSK	Breach of undertaking regarding misleading claim in material that implied superiority of Avandament over sulphonylureas in terms of glycemic control.	Breach of undertaking; Misleading claim
2012/6/07	Competact (pioglitazone/ metformin)	Takeda	Mailer incorrectly stated: "Unlike other glitazone combination(s) therapies, Competact costs LESS to prescribe than its constituent parts". Despite acknowledging in inter-company dialogue that the claim was misleading, Takeda had continued to make use of claim.	Misleading claim

Table S5 cont'd. Serious violations: promotion of diabetes drugs in the UK

Case no.	Drug	Company	Description	Violation
2044/9/07	Insulin	Eli Lilly	Without apparent company approval, a representative implied that continued funding of an educational post within the local diabetes network could be in danger if the hospital did not increase its use of Lilly insulins.	Conduct of representative
2125/5/08	Actos (pioglitazone)	Takeda	Advertisement claimed: "There are no long-term cardiovascular concerns regarding the use of Actos". Failed to mention that the drug might exacerbate and precipitate heart failure and was contraindicated in patients with, or with a history of, heart failure.	Misleading claim; Off-label promotion
2202/1/09	Victoza (liraglutide)	Novo Nordisk	Novo Nordisk promoted Victoza in a newspaper supplement (The Times) on World Diabetes day and, further, did so prior to the granting of marketing authorization.	Promotion to the public; Pre-licensing promotion
2269/9/09	Victoza (liraglutide)	Novo Nordisk	Breach of undertaking regarding case 2002/1/09. Novo Nordisk made the journal supplement available on its website months after the ruling.	Breach of undertaking; Promotion to the public
2234/5/09	Victoza (liraglutide)	Novo Nordisk	Four §2 rulings regarding pre-licensing promotion of Victoza (Liraglutide; Novo Nordisk): on website; via sponsored meetings disguised as scientific and medical meetings; via paid-for insert in medical journal disguised as independent supplement; at diabetes meeting by professor who failed to disclose financial relationship with company.	Pre-licensing promotion (n=4); Disguised promotion (n=2)

Table S5 cont'd. Serious violations: promotion of diabetes drugs in the UK

Case no.	Drug	Company	Description	Violation
2310/4/10	Byetta (exenatide)	Eli Lilly	At a meeting convened by Lilly in conjunction with the 2010 Diabetes UK Annual Professional Conference, the unlicensed once-weekly formulation of exenatide was promoted.	Pre-licensing promotion
2366/10/10	Byetta (exenatide)	Eli Lilly	Lilly sales representatives took two Diabetes specialist nurses to a restaurant for dinner with alcoholic beverages. The level of hospitality was found "excessive".	Hospitality
2424/8/11 and 2425/8/11	Trajenta (linagliptin)	Boehringer Ingelheim	Article in Future Prescriber constituted disguised and pre-licensing promotion of linagliptin by Boehringer-Ingelheim. The article incorrectly claimed that the drug had received marketing authorization in the UK, and that it was "safer to use" together with some medications than saxagliptin despite the lack of head-to-head trials.	Disguised promotion; Pre-licensing promotion; Misleading claim
