# Supplementary material for: Complaints, Complainants, and Rulings Regarding Drug Promotion in the United Kingdom and Sweden 2004–2012: A Quantitative and Qualitative Study of Pharmaceutical Industry Self-Regulation
Source: PLoS Med. 2015 Feb 17;12(2):e1001785. doi: 10.1371/journal.pmed.1001785 (PMC4331559; doi:10.1371/journal.pmed.1001785)
Supplement: S6 Table — (PDF) [file pmed.1001785.s007.pdf]

Table S6. Serious violations: promotion of diabetes drugs in Sweden

Case no.	Drug	Company	Description	Violation
W623/04	Levemir (Insulin detemir)	Novo Nordisk	Novo Nordisk convened a symposium to promote Levemir before marketing authorization was granted.	Pre-licensing promotion
W647/04 and W648/04	Glucobay (acarbose)	Bayer	The claim in mailing and advertisements that Glucobay had “cardioprotective effect” in patients with impaired glucose tolerance (IGT) and Type 2 Diabetes constituted off-label promotion since the drug was approved neither for patients with IGT nor for any cardioprotective use.	Off-label promotion
W664/04	Levemir (Insulin detemir)	Novo Nordisk	Brochure and advertisement contained misleading claims that Levemir use results in “physiological blood sugar control”, “physiological insulin profile”, “smooth insulin profile”, less within-individual variability in glucose levels than other products, “consistent day-to-day effect” and “minimal day-to-day variability”.	Misleading claim
W691/05	Levemir (Insulin detemir)	Novo Nordisk	Breach of undertaking regarding case W664/04. During a meeting NovoNordisk handed out patient information material containing claims previously found misleading.	Breach of undertaking; Misleading claim
W969/08	Levemir (Insulin detemir)	Novo Nordisk	Mailing to doctors was entitled “Dosage once per day recommended for Levemir which also results in uniquely low weight-gain, according to new study”. However, the referred study actually reported that 55% needed Levamir twice a day, and concluded that the reduction in weight-gain was “modest” vs. comparable treatment.	Misleading claim

Table S6 cont'd. Serious violations: promotion of diabetes drugs in Sweden

Case no.	Drug	Company	Description	Violation
885/10	Victoza (liraglutide)	Novo Nordisk	Advertisement in medical journals conveyed the incorrect impression that the drug cured diabetes, and referred misleadingly to the SPC to support the unsubstantiated claim that "starting treatment early with Victoza gives significant and lasting advantages" including "improved beta-cell function".	Misleading claim
