# Supplementary material for: Complaints, Complainants, and Rulings Regarding Drug Promotion in the United Kingdom and Sweden 2004–2012: A Quantitative and Qualitative Study of Pharmaceutical Industry Self-Regulation
Source: PLoS Med. 2015 Feb 17;12(2):e1001785. doi: 10.1371/journal.pmed.1001785 (PMC4331559; doi:10.1371/journal.pmed.1001785)
Supplement: S7 Table — (PDF) [file pmed.1001785.s008.pdf]

Table S7. Serious violations: promotion of urologics in the UK

Case no.	Drug	Company	Description	Violation
1741/7/05	Levitra (vardenafil)	Bayer	Bayer invited health professionals to a one and a quarter hour meeting on "The medical and surgical management of erectile dysfunction" followed by a champagne reception and gourmet dinner. Invitation had sought to attract delegates by inviting to prestigious restaurant. Hospitality was found "excessive".	Hospitality
1888/9/06	Levitra (vardenafil)	Bayer	A leaflet displayed at the meeting of the British Association of Urological Surgeons contained a claim regarding the efficacy of Levitra previously found misleading.	Breach of undertaking; Misleading claim
2151/7/08 and 2163/8/08	Cialis (tadalafil)	Eli Lilly	As part of Lilly's disease-awareness campaign for erectile dysfunction (ED) – 40over40 – the company posted a treatment option chart on www.40over40.com (case 2163/8/08) and in a leaflet (case 2151/7/08). While not naming products the first entry of the chart was identifiable as Cialis, through a description of its characteristics. The efficacy of ED drugs was stressed, but there was very limited information on side effects or contraindications. The panel considered that Lilly had marketed Cialis in ways that would encourage patients to request a specific prescription only medicine, and had failed to give a balanced description of the positive and negative characteristics of the products.	Promotion to the public (n=2); Misleading claim (n=2)

Table S7 cont'd. Serious violations: promotion of urologics in the UK

Case no.	Drug	Company	Description	Violation
2333/7/10	Levitra (vardenafil)	Bayer	Mailing described Levitra as the "first choice for erectile dysfunction" and without qualification claimed that it was a "safe option" and had demonstrated "efficacy and safety". Mailing also claimed: "According to NICE guidance for Type 2 Diabetes vardenafil should therefore become the preferred prescribing option for erectile dysfunction". This implied that NICE had specifically recommended Levitra and that was not so. NICE recommended choosing the medicine with the lowest acquisition cost. During consideration of the case, the Appeal Board realized that Bayer had circulated the material more widely than previously indicated by the company.	Misleading claim
2402/4/11	Levitra (vardenafil)	Bayer	Bayer posted an uncertified tweet regarding the launch of a new formulation for Levitra. This was considered advertising to the public.	Promotion to the public
