# Supplementary material for: Complaints, Complainants, and Rulings Regarding Drug Promotion in the United Kingdom and Sweden 2004–2012: A Quantitative and Qualitative Study of Pharmaceutical Industry Self-Regulation
Source: PLoS Med. 2015 Feb 17;12(2):e1001785. doi: 10.1371/journal.pmed.1001785 (PMC4331559; doi:10.1371/journal.pmed.1001785)
Supplement: S8 Table — (PDF) [file pmed.1001785.s009.pdf]

Table S8. Serious violations: promotion of urologics in Sweden

Case no.	Drug	Company	Description	Violation
W593/04	Avodart (dutasteride)	GSK	Brochure contained statements and images (of doctors with outsized tools) that exaggerated the effect of the drug.	Misleading claim
W585/04	Cialis (tadalafil)	Eli Lilly	A leaflet was disseminated at a urology meeting containing the claim that 9 of 10 patients preferred Cialis over Viagra, which had previously been found misleading.	Breach of undertaking; Misleading claim
W591/04	Levitra (vardenafil)	Bayer	Claims in letter to doctors were found misleading and inconsistent with the SPC, including that Levitra was the optimal treatment for Erectile Dysfunction, with quicker and more reliable response than other products.	Misleading claim
W595/04	Levitra (vardenafil)	Bayer	Claims in a brochure were found misleading and inconsistent with the SPC, including that Levitra was the most potent PDE5-inhibitor, that 9 of 10 patients on standard dose reported an improved erection, that the drug created “novel opportunities for many men” (implying that other drugs did not), and that the drug was somehow “fast, reliable, simple”.	Misleading claim
W627/04	Viagra (sildenafil)	Pfizer	Website aimed at the public mentioned Viagra by name despite a previous ruling on the matter.	Breach of undertaking; Promotion to the public
W629/04	Levitra (vardenafil)	Bayer	Breach of undertaking (IGM W591/04 and IGM 595/04). Bayer continued to market Levitra with claims that were inconsistent with the SPC, e.g. that the drug could be taken only 10 min before sexual activity rather than 25 to 60 min.	Breach of undertaking; Misleading claim

Table S8 cont'd. Serious violations: promotion of urologics in Sweden

Case no.	Drug	Company	Description	Violation
W635/04	Cialis (tadalafil)	Eli Lilly	On a website aimed at health professionals, Lilly professed that "7 of 10 men with impotence chose Cialis before Viagra". To support this claim Lilly cited a study, but failed to note several limitations, such as that it compared the highest approved dose for Cialis to the lowest recommended dose for Viagra. IGM had on two occasions (including IGM W585/04) demanded that Lilly stop making unjust comparisons and cite data fairly.	Breach of undertaking; Misleading claim
W859/06	Vesicare (solifenacin)	Astellas	Claims in brochures misrepresented the published evidence and omitted important information. For example, it was claimed that Vesicare was "significantly better" than another product on 8 of 10 parameters, but no p values were provided and, in fact, for important parameters differences were minor and clinical relevance unclear. Also, brochures claimed that on 2 of 10 parameters drugs were "equal", but the cited study had tested for non-inferiority.	Misleading claim
W914/07	Vesicare (solifenacin)	Astellas	Breach of undertaking (IGM W859/06). Astellas continued to market Vesicare by claiming that it was "significantly better" than another product on 8 of 10 parameters without providing p values and effect sizes.	Breach of undertaking; Misleading claim

Table S8 cont'd. Serious violations: promotion of urologics in Sweden

Case no.	Drug	Company	Description	Violation
W950/07 and W955/07	Levitra (vardenafil)	Bayer	Breach of undertaking (IGM W591/04, IGM 595/04 and IGM 629/04). Bayer continued to market Levitra with claims of rapid onset inconsistent with the SPC despite three successive rulings on this matter.	Breach of undertaking; Misleading claim
W958/07	Cialis (tadalafil)	Eli Lilly	Advertisement was found misleading on several points, including claims that ED patients and their partners preferred Cialis over Viagra and that the drug made "a real difference" (implying that other drugs did not). A study was cited to support claim that patients preferred Cialis because it gave them "harder erections" and "36-hours duration of effect", but the study did not support claims.	Misleading claim
785/07	Levitra (vardenafil)	Bayer	Brochure for Levitra that described a double-blind RCT comparing Levitra and Viagra misrepresented the study's conclusions. In particular, Bayer failed to include numerical values on relevant outcomes and to clearly differentiate between primary and secondary outcome measures.	Misleading claim
913/11	Vesicare (solifenacin)	Astellas	Astellas sent letter entitled "Important information for people who work in health care" to doctors. The label "Important information" is only allowed for new information on ADR, contraindications, restrictions and withdrawals. The letter contained no such information, but instead promoted Vesicare. The MPA alleged disguised promotion, but the NBL rejected the allegation on this point.	Rules on information
