# Supplementary material for: Complaints, Complainants, and Rulings Regarding Drug Promotion in the United Kingdom and Sweden 2004–2012: A Quantitative and Qualitative Study of Pharmaceutical Industry Self-Regulation
Source: PLoS Med. 2015 Feb 17;12(2):e1001785. doi: 10.1371/journal.pmed.1001785 (PMC4331559; doi:10.1371/journal.pmed.1001785)
Supplement: S1 Text — (DOCX) [file pmed.1001785.s010.docx]

**Text S1. Additional information on the qualitative coding and analysis process**

Documents and reports were analyzed using qualitative content analysis to outline similarities and differences between systems regarding their constitution and the procedures, under which the materials and activities of pharmaceutical companies are considered in relation to the requirements of the Code. Content analysis refers here to a qualitative data reduction and sense-making effort that takes a volume of qualitative material and attempts to identify core consistencies. The procedure is described in [1]. Briefly, it was as follows. Data were collected and divided into meaning units, i.e. sentences or paragraphs containing aspects related to each other through their content and context. Meaning units were condensed. Condensed meaning units were abstracted and coded into categories and when relevant into sub-categories. Our analysis centered on the respective Codes and associated documents outlining the workings of the systems. These were read thoroughly several times in order to get an understanding of their structure and content. We also read reports produced by self-regulatory bodies on an annual, biannual or quarterly basis, as well as numerous individual case reports issued by self-regulatory bodies, to get an understating of the actual working of systems. The sections of the documents that pertained to the constitution and procedures of self-regulatory systems were coded into categories and sub-categories and then read again, which resulted in some new categories and sub-categories. At this point we also compared the categories, sub-categories and meaning units obtained from the two countries, which resulted in additional categories and sub-categories. An example of the coding and analysis process is presented in the Table below.

| **Meaning Unit^1^** | **Condensed meaning unit** | **Category** | **Sub-category** |
| --- | --- | --- | --- |
| The task of monitoring the market is handled by the IGM. | The IGM undertakes active monitoring | Active monitoring by self-regulatory bodies | Responsible body |
| In order for the IGM to be able to carry out this task, the pharmaceutical companies must send new, up-to-date drug information to the IGM, such as publications, advertisements, invitations, mailings, commercial films or information on websites. | Companies required to submit material |  | Material collection |
| *The Authority arranges for the scrutiny of samples of advertisements, detail aids, leavepieces, other promotional items and meetings on a continuing basis in relation to the requirements of the Code* | The PMCPA undertakes active monitoring |  | Responsible body |
| *To facilitate such scrutiny, the Director may request relevant material from pharmaceutical companies* | Companies submit material upon request |  | Material collection |
| Competing pharmaceutical companies that submit a report to the IGM or NBL must enclose evidence that the company or companies against whom the representation is made has been encouraged to terminate or change the criticised measure but has/have failed to observe such request within two weeks of receiving it. | Companies must first attempt to settle disagreements through inter-company dialogue | Inter-company dialogue | Requirement |
| *A complaint from a pharmaceutical company will be accepted only if the Director is satisfied that the company concerned has previously informed the company alleged to have breached the Code that it proposed to make a formal complaint and offered inter-company dialogue at a senior level in an attempt to resolve the matter, but that this offer was refused or dialogue proved unsuccessful. A formal statement detailing the actions taken must be provided* | Companies must first attempt to settle disagreements through inter-company dialogue |  | Requirement |

^1^ Normal fonts: Sweden; Italics font: UK.

**References**

1. Graneheim UH, Lundman B (2004) Qualitative content analysis in nursing research: concepts, procedures and measures to achieve trustworthiness. Nurse Educ Today 24:105-112.
